# Repeated Phenotypic Evolution by Different Genetic Routes in *Pseudomonas fluorescens* SBW25

**DOI:** 10.1093/molbev/msz040

**Published:** 2019-03-05

**Authors:** Jenna Gallie, Frederic Bertels, Philippe Remigi, Gayle C Ferguson, Sylke Nestmann, Paul B Rainey

**Affiliations:** 1 Department of Evolutionary Theory, Max Planck Institute for Evolutionary Biology, Plön, Germany; 2 New Zealand Institute for Advanced Study, Massey University at Albany, Auckland, New Zealand; 3 Department of Microbial Population Biology, Max Planck Institute for Evolutionary Biology, Plön, Germany; 4 Laboratoire des Interactions Plantes-Microorganismes (LIPM), Université de Toulouse, INRA, CNRS, Castanet-Tolosan, France; 5 School of Natural and Computational Sciences, Massey University at Albany, Auckland, New Zealand; 6 Ecole Supérieure de Physique et de Chimie Industrielles de la Ville de Paris (ESPCI ParisTech), CNRS UMR 8231, PSL Research University, Paris, France

**Keywords:** evolution, genetics, microbiology

## Abstract

Repeated evolution of functionally similar phenotypes is observed throughout the tree of life. The extent to which the underlying genetics are conserved remains an area of considerable interest. Previously, we reported the evolution of colony switching in two independent lineages of *Pseudomonas fluorescens* SBW25. The phenotypic and genotypic bases of colony switching in the first lineage (Line 1) have been described elsewhere. Here, we deconstruct the evolution of colony switching in the second lineage (Line 6). We show that, as for Line 1, Line 6 colony switching results from an increase in the expression of a colanic acid-like polymer (CAP). At the genetic level, nine mutations occur in Line 6. Only one of these—a nonsynonymous point mutation in the housekeeping sigma factor *rpoD*—is required for colony switching. In contrast, the genetic basis of colony switching in Line 1 is a mutation in the metabolic gene *carB*. A molecular model has recently been proposed whereby the *carB* mutation increases capsulation by redressing the intracellular balance of positive (ribosomes) and negative (RsmAE/CsrA) regulators of a positive feedback loop in capsule expression. We show that Line 6 colony switching is consistent with this model; the *rpoD* mutation generates an increase in ribosomal gene expression, and ultimately an increase in CAP expression.

## Introduction

The repeated appearance of similar phenotypes is a striking feature amid the diversity of life. Many phenotypes have evolved multiple independent times in different lineages (Conway Morris 1999). Examples include the evolution of analogous wing-like structures for flight in pterosaurs, birds, insects, and bats (Alexander 2015), C4 photosynthetic pathways in plants (Sage et al. 2011), and single-lens camera eyes in vertebrates and molluscs (Ogura et al. 2004). An intriguing aspect of repeated phenotypic evolution is the extent to which the underlying genetics are also conserved. It is commonly thought that the degree of genetic parallelism correlates with the degree to which two organisms are related. The accumulation of genetic data in recent years has shown this assumption to be in need of revision. For example, clonal populations of *Escherichia coli* adapt to thermal stress via different genetic routes (Riehle et al. 2001), whereas pigmentation changes in mice and lizards are both underpinned by mutations in the *Mc1r* gene (Nachman et al. 2003; Rosenblum et al. 2004). The increasing number of examples of disparity between genetic parallelism and degree of relatedness (reviewed in Arendt and Reznick 2008) hints at the underappreciated and poorly understood complexity of biological systems.

**Fig. 1.**
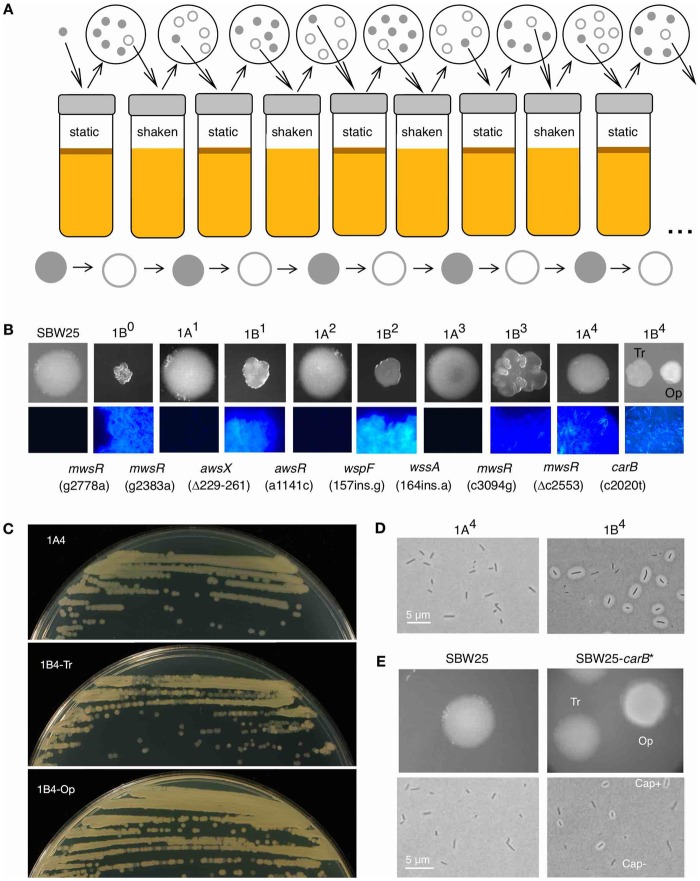
Emergence of colony switching in reverse evolution experiment (REE) Line 1. (*A*) Cartoon of one Line of the 12-Line REE (Beaumont et al. 2009). Populations were subjected to bouts of selection in shaken (environment A) or static (environment B) liquid KB. After each bout, cells were plated on KB agar and a colony with novel morphology was purified and used to start the next round in the opposite environment. (*B*) Line 1 phenotypes and genotypes. Each Line 1 strain produces colonies distinct from those of its immediate ancestor (first row). Strains differ in their ability to produce cellulose (cells grown on KB+calcofluor agar; second row). Mutations are noted as “*gene* (mutation)” at the point of fixation. (*C*) Bi-directional colony switching in 1B^4^. 1A^4^ generates colonies of a single type (top), whereas 1B^4^-Tr (middle) or -Op colonies (bottom) generate a mixture. (*D*) 1B^4^ cells are capsulated (Cap^+^) or noncapsulated (Cap^−^); 1A^4^ cells are generally Cap^−^. (*E*) SBW25 colonies are uniform whereas SBW25-carB* (SBW25 into which the c2020t *carB* mutation is engineered) shows colony bistability. Colonies grown on KB agar (28 °C, 48 h); cells grown in shaken KB (16–24 h) before staining and bright field or fluorescence microscopy. Exposure of some images altered in Preview. Tr, translucent; Op, opaque.

An evolution experiment with populations of the model bacterium *Pseudomonas fluorescens* SBW25 (Beaumont et al. 2009) has provided an opportunity to characterize a case of repeated phenotypic evolution in unusual detail. Twelve independent populations were subjected to multiple rounds of selection for novel colony morphology. Each round of selection concluded with the isolation of a single colony per population. This colony had a phenotype different from that of the immediate ancestor, and was used to found the subsequent round of selection (fig. 1*A*). The final result was 12 independent evolutionary lineages, each with a clearly defined history of colony phenotypes and underlying genetic changes. Two lineages (Line 1 and Line 6) converged on a striking capacity to stochastically switch between two different colony morphologies at high frequency.

Colony switching in Line 1 has been extensively investigated (Beaumont et al. 2009; Libby and Rainey 2011; Rainey et al. 2011; Gallie et al. 2015; Remigi et al. 2019). Emergent genotype 1B^4^ produces a mixture of opaque and translucent colonies, and a corresponding mixture of capsulated and noncapsulated cells (fig. 1*B* and *C*; Beaumont et al. 2009). The capsule consists of a colanic acid-like polymer (CAP), the ON/OFF expression of which leads to colony switching (Gallie et al. 2015). Nine mutational steps occurred during the evolution of 1B^4^ (fig. 1*B*). The first eight occur in genes involved in the production of c-di-GMP, a secondary messenger that affects the expression of an acetylated cellulosic polymer (cellulose for short; Spiers et al. 2003; McDonald et al. 2009, fig. 1*B*). The final mutation affected the central metabolic gene *carB* (c2020t, giving amino acid change R674C). This mutation, which is alone sufficient to cause colony switching (fig. 1*D*), perturbs intracellular pyrimidine pools (Gallie et al. 2015). Pyrimidine deficiency in 1B^4^ has recently been shown to generate—by a currently unknown mechanism—an increase in intracellular ribosome concentration (Remigi et al. 2019). This has led to the proposal of a translational control model for capsule switching (Remigi et al. 2019). Briefly, the model proposes that capsule switching results from competition for binding sites on the mRNA of *pflu3655–pflu3657*, which encodes transcriptional regulators of CAP biosynthetic genes; ribosome binding results in translation (and promotion of capsulation), whereas RsmAE/CsrA binding inhibits translation (favoring the noncapsulated state). The ribosome increase in 1B^4^ tips the balance of the switch in favor of translation, increasing the probability of capsulation.

In this work, we characterize the phenotypic and genetic bases of colony switching in the second emergent genotype, 6B^4^. Comparisons with 1B^4^ demonstrate that 6B^4^ colony switching is a very similar phenotype realized by a different genetic route. We also show that the two genetic routes are reconciled at the molecular mechanistic level.

## Results

### 6B^4^ Shows Colony and Capsule Instability

**Fig. 2.**
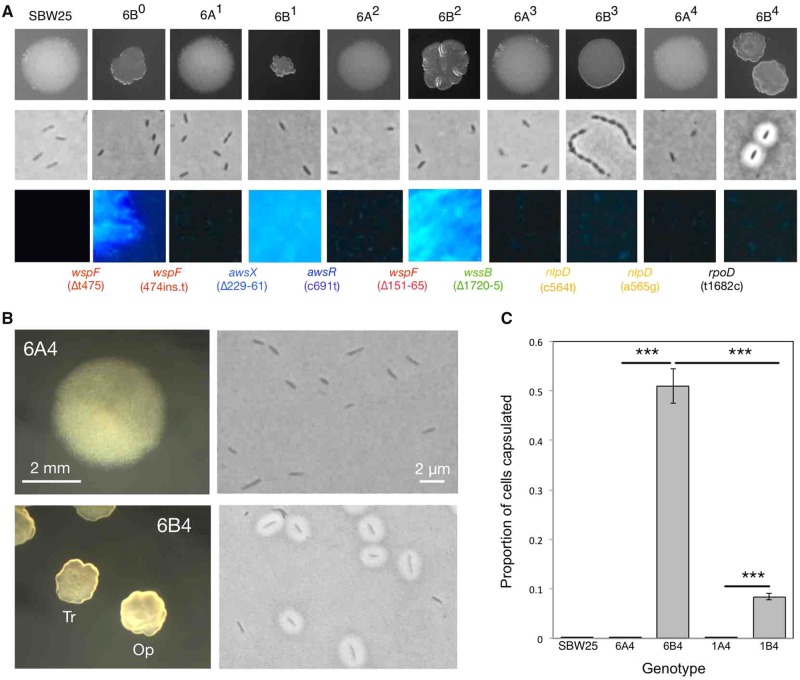
Emergence of colony switching in REE Line 6. (*A*) Phenotypes and genotypes of Line 6: colony morphology on KB agar (row 1), morphology of cells grown in shaken KB microcosms and stained with India ink (row 2), ability of cells grown on KB agar with calcofluor to produce cellulose (row 3), and mutations (shown at point of occurrence; bottom). (*B*) Colony and cell morphologies of 6B^4^ and its nonswitching immediate ancestor, 6A^4^. 6B^4^ gives rise to translucent (Tr) and opaque (Op) colonies, plus capsulated and noncapsulated cells. (*C*) The proportion of capsulated cells in various populations during stationary phase. Each bar is the mean of five replicate populations grown overnight in KB microcosms. Error bars are one standard error and stars denote statistical significance (***P < 0.001). Contrast and/or exposure of some images altered in Preview.

The evolutionary history of 6B^4^ includes ten colony phenotypes, with translucent–opaque colony instability emerging after nine rounds of selection (fig. 2*A*). 6B^4^ colonies comprised a mixture of capsulated and noncapsulated cells, and 6B^4^ populations contain a significantly higher proportion of capsulated cells than those of the immediate ancestor, 6A^4^ (fig. 2*B* and *C*; Welch two-sample *t-*test *P* = 1.3 × 10^−4^). Single 6B^4^ cells of either type give rise to mixed Cap^+^/Cap^−^ populations (supplementary text S1, Supplementary Material online). We conclude that colony switching in 6B^4^ has the same underlying phenotypic basis as in 1B^4^: the ON/OFF switching of capsule biosynthesis. However, under the conditions tested, the proportion of capsulated cells is significantly higher in 6B^4^ than in 1B^4^ populations (fig. 2*C*; Welch two-sample *t*-test 1.9 × 10^−4^). In addition, on KB agar 6B^4^ capsules are 1.26–1.83 times larger than those in 1B^4^ (two sample *t*-test for no difference in capsule area *P* = 9.602 × 10^−10^), despite no difference in cell size (two sample *t-*test for no difference in cell area *P* = 0.5236; see supplementary text S1, Supplementary Material online).

### 6B^4^ Capsule Expression Is Due to Transcriptional Regulation of *wcaJ–wzc*

To identify the genetic basis of the 6B^4^ capsule, 6B^4^ was subjected to transposon mutagenesis. In a screen of ∼10,000 transposon mutants, 55 with altered levels of capsulation were identified, and the transposon insertion site determined for each (supplementary table S1, Supplementary Material online). Microscopic screening of cells showed capsule production to be eliminated in 43 genotypes, and severely reduced in a further nine genotypes. Three genotypes showed an increase in capsule production.

Of the genotypes with eliminated or reduced capsule production, 41 (75%) contained insertions in genes required for the production of a CAP, a polymer previously described as the structural basis of the 1B^4^ capsule (Gallie et al. 2015). These include insertions in genes predicted to encode CAP precursor biosynthetic machinery (e.g., *algC*), CAP biosynthetic machinery (20 genes: *wcaJ–wzc*) and CAP regulators (*pflu3655*, *pflu3656*, *pflu3657*, *gacA/gacS*). A direct deletion of the CAP biosynthetic locus from 6B^4^ resulted in loss of both cell capsulation and colony bistability (supplementary fig. S1, Supplementary Material online). Together these results demonstrate that the structural basis of the 6B^4^ capsule is encoded by the *wcaJ–wzb* locus.

To investigate whether CAP production is controlled at the level of transcription, transcriptional fusions were constructed in 6A^4^ and 6B^4^; *lacZ* was transcriptionally fused to *wcaJ* (*pflu3658*), the first gene in the CAP biosynthetic locus (supplementary text S2; see Supplementary Material online for details). Cotranscription of *wcaJ* and *lacZ* in these strains generates blue colonies (or blue sectors within white colonies) on LB agar supplemented with X-gal. Indeed, 6B^4^-*wcaJ*–*lacZ* produced a mixture of white and blue colonies (supplementary fig. S1, Supplementary Material online), with high proportions of Cap^−^ and Cap^+^ cells, respectively. The same construction in 6A^4^—the immediate switch ancestor—resulted in uniform, nonsectored colonies. Together, these results show that CAP expression is at least partially controlled at the level of transcription (later corroborated by RNA-seq data; supplementary tables S3–S5; see Supplementary Material online for details).

### The Structural Basis of the 6B^4^ Capsule Is CAP

To directly investigate composition of the 6B^4^ capsule, extracellular polysaccharide (EPS) was extracted from SBW25, 6A^4^, 6B^4^, 1A^4^, and 1B^4^, and the component sugars from each strain analyzed by chromatography (results for SBW25, 1A^4^, 1B^4^ reported previously in Gallie et al. 2015; supplementary fig. S1 and table S2, Supplementary Material online). The analysis shows differences in the expression of several components: in 6B^4^ relative to 6A^4^, the expression of d-fucose (Fuc), d-glucuronic acid (GlcA), d-galacturonic acid (GalA), and two unknowns are increased. Each of these is also increased in 1B^4^ relative to 1A^4^, indicating that the 1B^4^ and 6B^4^ capsule polymers are very similar.

Thus far, the transposon mutagenesis, strain constructions and structural analysis of the capsule polymers (and later, RNA-seq data) point to the same phenotype for 1B^4^ and 6B^4^: switching between opaque and translucent colonies caused, at the single cell level, by ON/OFF expression of CAP. The only difference observed between the two genotypes lies in the frequency of capsulation and size of capsules (both increased in 6B^4^ relative to 1B^4^).

### The Mutational History of 6B^4^

Next, the genetic basis of 6B^4^ capsule switching was investigated. Whole genome sequencing of 6B^4^ identified seven mutations. This was surprising, as at least nine mutations were expected - one per round of reverse evolution experiment (REE) selection (see fig. 1*A*). Sanger sequencing across the evolutionary series revealed two gaps: SBW25→6B^0^ (selection round 1) and 6B^0^→6A^1^ (selection round 2; table 1, fig. 2*A*). Extensive previous knowledge suggested that these two genotypes almost certainly carried mutations in one of three loci (*wsp*, *aws*, *mwsR*; McDonald et al. 2009). Sanger sequencing of *wspF* revealed a point deletion in 6B^0^ (Δt475) that was reversed in 6A^1^, rendering 6A^1^ isogenic to the wild type SBW25 (fig. 2*A*). The reversal event was not repeated among 20 independent replicates of a single round of REE from 6B^0^, meaning that 6A^1^ is the result of either a rare mutational event, or recovery of SBW25 from previous rounds.

**Table 1.** Mutations in the Line 6 Evolutionary Series.

Strain	Gene		Nucleotide Change	Amino Acid Change	Morph^c^
	*pflu*	Name			
SBW25	—	—	—	—	Smooth
6B^0^	1224	*wspF*	Δt475	ΔS159(40)^a^	Wrinkly
6A^1^	1224	*wspF*	474ins.t	S158ins(178)^b^	Smooth
6B^1^	5211	*awsX*	Δ229–261	Δ77–87(ΔYTDDLIKGTTQ)	Wrinkly
6A^2^	5210	*awsR*	c691t	Q231STOP	Smooth
6B^2^	1224	*wspF*	Δ151–165	Δ51–55(ΔLMDLI)	Wrinkly
6A^3^	0301	*wssB*	Δ1720–1725	Δ574–575(ΔVA)	Smooth
6B^3^	1301	*nlpD*	c565t	Q189STOP	Round
6A^4^	1301	*nlpD*	a566g	STOP189W	Smooth
6B^4^	5592	*rpoD*	t1682c	V561A	Switcher

a ΔS159(40) indicates a frame shift caused by a base deletion; number of new residues before a stop codon is in parentheses.

b S158ins(178) indicates a frame shift by a base insertion; number of new residues prior to a stop codon is in parenthesis.

c Morph indicates phenotype on the basis of colony morphology on KB agar and cellulose production.

The Line 6 mutations occur in a modular, paired fashion. The first six mutations occur in previously identified c-di-GMP producing loci (*awsX/awsR*, *wspF/wssB*); mutations in these loci are known to cause the gain and loss of cellulose production and wrinkly spreader colony morphology (Beaumont et al. 2009; McDonald et al. 2009; Gallie et al. 2015; Lind et al. 2015, 2017; Lind PA, Libby E, Herzog J, Rainey PB, 2019). The sixth mutation—an in-frame, six bp deletion in the cellulose biosynthetic gene *wssB*— -->completely abolishes cellulose production (fig. 2*A*). Accordingly, the next pair of mutations occur in an unrelated locus: *nlpD* (*pflu1301*), which encodes a lipoprotein predicted to have a function in cell wall formation and cell separation in a range of bacteria (Stohl et al. 2016; Lind et al. 2017; Tsang et al. 2017; Yang et al. 2017). The first of these, mutation seven, generates a nonsense mutation in *nlpD* resulting in the production of cell chains and round colonies in 6B^3^ (fig. 2*A*). This mutation has previously been reported to generate a cell chain phenotype in SBW25 (Lind et al. 2017), and similar mutations have been reported in *E. coli* (Uehara et al. 2010), *Vibrio cholerae* (Möll et al. 2014), and *Yersinia pestis* (Tidhar et al. 2009). In short, NlpD is an activator of cell division protein AmiC; inactivation of NlpD leads to incomplete cell division. Mutation eight converts the *nlpD* nonsense mutation into a tryptophan residue, reversing the cellular and colony phenotypes (fig. 2*A*). The final mutation, with which colony switching emerges, is in *rpoD* (t1682c, resulting in amino acid change V561A). This gene encodes the housekeeping sigma factor (σ^70^) that controls transcription of many genes involved in cell growth and division (Schulz et al. 2015).

There are two notable points of similarity and contrast between the evolutionary histories of 6B^4^ (fig. 2*A*) and 1B^4^ (fig. 1*B*). First, both lineages begin in a similar fashion with mutations affecting cellulose production and wrinkly spreader colony morphology. In Line 6, mutational routes to the wrinkly spreader phenotype are presumably rendered inaccessible by the sixth mutation (in *wssB*), providing an opening for a pair of mutations in *nlpD*. Contrastingly, cellulose production is not abolished in Line 1, with 1B^4^ staining positive for cellulose (Gallie et al. 2015, fig. 1*B*). Accordingly, Line 1 mutations are in cellulose-affecting loci up until the final, switch-causing mutation. Second, the final mutation in each lineage—that with which colony switching emerges—is a nonsynonymous point mutation in different and, at first glance, functionally unrelated housekeeping genes.

### The *rpoD* t1682c Mutation Alone Generates an Increase in Capsulation

**Fig. 3.**
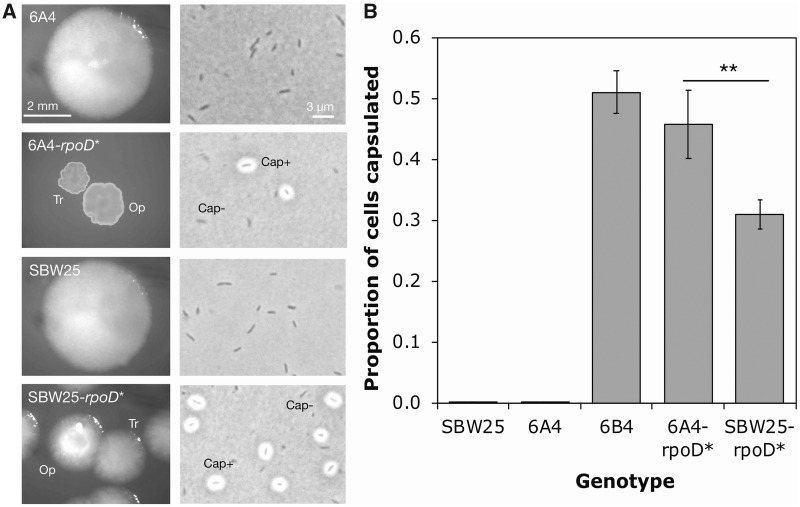
The t1682c *rpoD* mutation causes the emergence of capsule switching in both the presence and absence of the other Line 6 mutations. (*A*) Engineered strains carrying the *rpoD* mutation develop a mixture of Tr/Op colonies on KB agar after 48 h, and a mixture of Cap^+^/Cap^−^ cells (cells grown overnight in KB glass microcosms and stained with India ink before bright field microscopy). Saturation and brightness of some photographs altered in Preview. (*B*) The proportion of capsulated cells in populations of various genotypes during stationary phase. Each bar represents the mean of five replicate populations grown overnight in KB glass microcosms. Error bars represent one standard error and stars show statistical significance (**0.01 < *P* < 0.001).

To confirm that the final mutation causes colony switching in the presence of the prior mutations, t1682c *rpoD* was engineered into the immediate ancestor, giving 6A^4^-rpoD* (supplementary text S2, Supplementary Material online). 6A^4^-rpoD* gives rise to high-level colony and CAP switching, showing the same proportion of capsulated cells as 6B^4^ (two sample *t*-test *P* = 0.45; fig. 3). The *rpoD* mutation was then engineered into the distant ancestor, SBW25, in the absence of any other mutations. The resulting genotype, SBW25-rpoD*, also showed distinct colony types and a high level of capsulation. A capsule counting assay revealed that whereas the *rpoD* mutation alone was sufficient to cause switching, SBW25-rpoD* showed a lower degree of capsulation than 6B^4^ (two sample *t*-test *P* = 1.6 × 10^−3^; fig. 3*B*). Therefore, whereas the *rpoD* mutation does cause CAP switching, one or more of the prior mutations—or additional mutations that were not identified by analysis of the genome sequencing data—contribute(s) quantitatively to 6B^4^ capsule switching. This is in contrast to the c2020t *carB* mutation in Line 1, which alone accounts for 1B^4^ capsule switching (Gallie et al. 2015).

### Repeated Evolution of Switcher Genotypes Reveals Additional *rpoD* Mutations

**Fig. 4.**
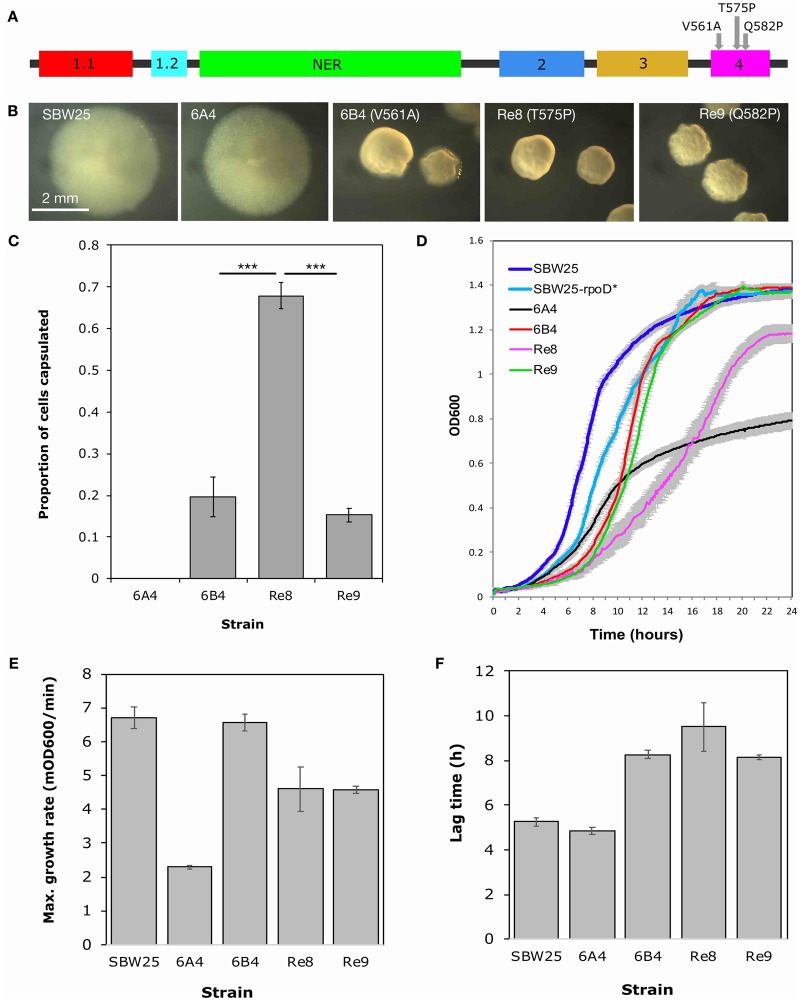
Three *rpoD* mutations have different effects on capsulation and growth. Three amino acid substitutions leading to switching have been identified in σ^70^. Each of these changes occurs in region 4, which encodes an H–T–H motif that binds to the −35 consensus sequence of σ^70^-dependent promoters.Nonessential region (*A*). In each case, the amino acid substitution leads to the emergence of two colony phenotypes or sectored colonies (*B*; colonies grown on KB agar for ∼56 h) and an increase in capsulation during a capsule counting assay in stationary phase (*C*; bars are the mean of five replicates). (*D*) Twenty-four-hour growth curves in shaken KB medium at 28 °C. Measurements were taken every 5 min, with eight replicates for each strain (against KB blanks). Mean maximum growth rates (*E*) and lag times (*F*) were calculated using a sliding window of six data points. Error bars on all graphs how one standard error.

To identify additional mutations able to cause capsule switching in 6A^4^, new switcher genotypes were evolved from 6A^4^. Each of 56 independent microcosms was inoculated with 6A^4^ and put through a single round of the REE (Beaumont et al. 2009). Nine new switcher genotypes were isolated from nine independent microcosms (genotypes Re1–Re9; supplementary text S1 and S2, Supplementary Material online). Sequencing of *rpoD* revealed a single, nonsynonymous point mutation in each; eight of the new switchers (Re1–Re8) contain mutation a1723c leading to amino acid change T575P, whereas one (Re9) carries a1745c causing amino acid change Q582P. All three *rpoD* mutations (t1682c, a1723c, a1745c) are located in the H–T–H motif that interacts with the −35 consensus sequence of σ^70^ dependent promoters (Hu and Gross 1988; Siegele et al. 1989, fig. 4*A*). Interestingly, mutation a1723c leads to a significantly higher capsulation rate than the others (two sample *t*-tests *P* < 0.001; fig. 4*B* and *C*).

Changes in a gene as central as *rpoD* are expected to have major effects on cell growth. Indeed, the growth profiles of each *rpoD* mutant differ from those of the ancestral strains (fig. 4*D*). The *rpoD* mutations increase growth rate and final density—at the cost of a longer lag phase—in comparison with 6A^4^ (fig. 4*D*–*F*). These results are consistent with the *rpoD* mutations affecting σ^70^ activity during exponential growth.

### Epistatic Interactions in Line 6 and Line 1

**Fig. 5.**
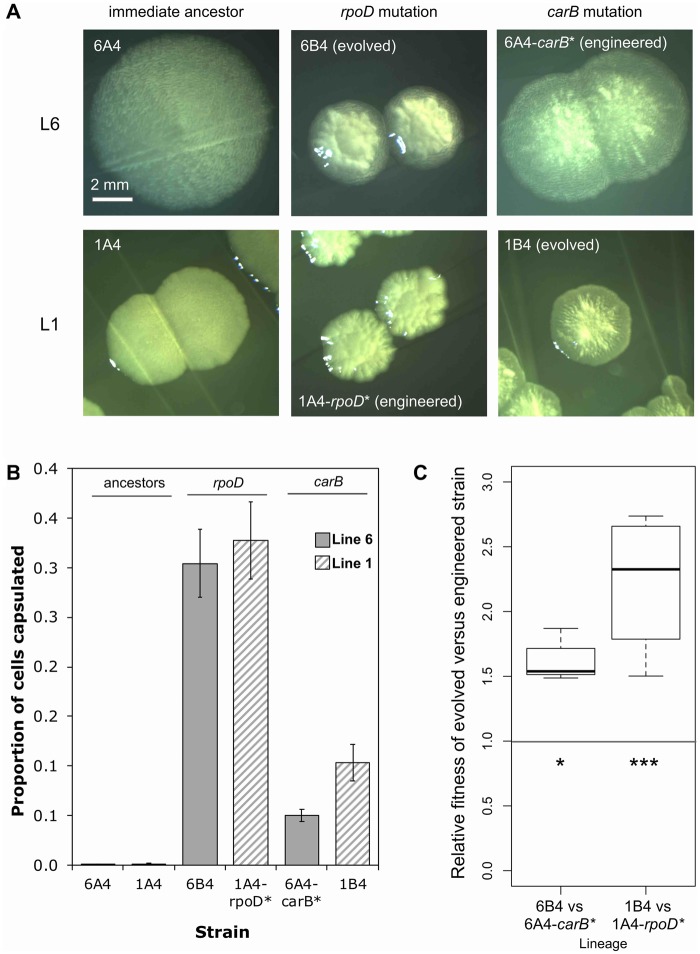
The fitness effect of switch-causing *rpoD* and *carB* mutations is lineage dependent. (*A*) The t1682c *rpoD* and c2020t *carB* mutations both cause colony switching in the 6A^4^ and 1A^4^ backgrounds (seen as two colony types and/or sectors on KB agar, 72 h). (*B*) A capsule counting assay in stationary phase shows that the *rpoD* and *carB* mutations cause an increase in capsulation relative to the immediate ancestor in both lineages (Wilcoxon rank sum tests *P* = 0.02857*). Bars = mean of four replicates, error bars are one SE. (*C*) Box plots of the fitness of the evolved type versus the engineered type in Line 6 and Line 1. Competition assays (1:1) were performed under the REE conditions in which the evolved types were originally isolated (72-h static microcosms). Values greater than 1 indicate a higher relative fitness of the first competitor (evolved types). Both competitions show a significant deviation from 1 (one-sided one sample *t*-test *P < 0.05, ***P < 0.001).

Nine independent switchers isolated from 6A^4^ each carried a point mutation in *rpoD* (see above and fig. 4*A*). Six independent switchers isolated from 1A^4^ each carried a point mutation in a pyrimidine biosynthetic gene (five in *carB*, one in *pyrH*; Gallie et al. 2015). To investigate this striking degree of lineage-dependent repeated evolution, the *rpoD* and *carB* mutations were swapped into the opposing backgrounds. That is, t1682c *rpoD* (from 6B^4^) was engineered into 1A^4^ (giving 1A^4^-rpoD*) and c2020t *carB* (from 1B^4^) was engineered into 6A^4^ (giving 6A^4^-carB*; supplementary text S2, Supplementary Material online). The engineered genotypes showed characteristic switcher colony morphologies (fig. 5*A*) and an increase in capsulation (fig. 5*B*; Wilcoxon rank sum tests *P* = 0.02857), demonstrating that the *rpoD* and *carB* mutations cause switching in both genetic backgrounds. Notably the *rpoD* mutation causes a significantly higher capsulation rate than the *carB* mutation in both Line 6 and Line 1 (fig. 5*B*; one-sided Wilcoxon rank sum test *P* = 0.01429 and one-sided two sample *t*-test *P* = 9.7 × 10^−4^, respectively). Next a competition experiment was performed under REE conditions between the evolved and engineered types from each Line (i.e., 6B^4^ vs. 6A^4^-carB* and 1B^4^ vs. 1A^4^-rpoD*). In each case, the evolved genotype outcompeted the engineered genotype—the *rpoD* mutation is fitter than the *carB* mutation in 6A^4^, and vice versa in 1A^4^ (fig. 5*C*). These results demonstrate epistatic interactions between switch-causing and prior mutation(s) in each Line.

### Gene Expression Differences in the Presence of the t1682c *rpoD* Mutation (RNA-seq)

Changes in the σ^70^-promoter recognition and binding domain are expected to affect expression from σ^70^-dependent promoters (or a subset thereof). Thus, the effect of the t1682c *rpoD* mutation on intracellular mRNA pools was investigated. Total mRNA was isolated from three biological replicates of exponentially growing 6A^4^, 6B^4^-Cap^−^, and 6B^4^-Cap^+^. RNA-seq was performed on the mRNA fraction, and three comparative analyses were generated: (A) 6A^4^ versus 6B^4^-Cap^−^, (B) 6A^4^ versus 6B^4^-Cap^+^, and (C) 6B^4^-Cap^−^ versus 6B^4^-Cap^+^. A list of genes with detectable expression levels (∼98% of all predicted genes in the SBW25 genome [Silby et al. 2009]) was generated for each comparison, and the three lists were then further split into genes with and without statistically significantly different expression levels (supplementary tables S3–S5, Supplementary Material online).

The greatest number of genes showing statistically significantly different expression was found in comparison B, 6A^4^ versus 6B^4^-Cap^+^, indicating that these are the two most physiologically distinct morphotypes. Of the 1,438 genes identified, 612 were more highly expressed in the ancestral 6A^4^ (including 33 flagella biosynthetic genes), and 826 were more highly expressed in 6B^4^-Cap^+^ (including 24 CAP and seven alginate biosynthetic genes). Comparison A, 6A^4^ versus 6B^4^-Cap^−^, identified 495 differentially expressed genes with statistical significance, 427 (86%) of which are shared with comparison B. Comparison of 6B^4^-Cap^−^ and 6B^4^-Cap^+^ identified 82 significantly differently expressed genes, 52 of which are more highly expressed in 6B^4^-Cap^−^ (including 30 flagella biosynthetic genes) and 30 in 6B^4^-Cap^+^ (including 12 CAP genes). Notably, mutant *rpoD* was found to be ∼1.74 times more highly expressed in 6B^4^-Cap^+^ than the wild type *rpoD* counterpart in 6A^4^ (adjusted *P* = 0.0324) indicating that the t1682c *rpoD* mutation leads to activation of *rpoD* transcription and/or inhibition of mRNA degradation (supplementary table S4, Supplementary Material online). Levels of *rpoD* mRNA in 6B^4^-Cap^−^ are intermediate between those in 6A^4^ and 6B^4^-Cap^+^, as no significant difference in *rpoD* mRNA levels was detected between 6B^4^-Cap^−^ and either of the other two types (supplementary tables S3 and S5, Supplementary Material online). A further five putative sigma factors are more highly expressed in 6B^4^-Cap^+^ than in 6A^4^ (*rspL*, *pflu2609*, *pflu2725*, *pflu3898*, *pflu4613*), indicating a general shift in gene expression.

A direct comparison of the changes in gene expression resulting from the *rpoD* (Line 6) and *carB* (Line 1) mutations may provide insight into molecular similarities between the strains. The equivalents of the above comparisons have been previously published for Line 1 (GEO GSE48900; Gallie et al. 2015). Although the numbers of differentially expressed genes are much higher in the Line 1 comparisons—most likely attributable to there being only a single biological replicate for each Line 1 morphotype—the overall pattern remains; the highest number of differentially expressed genes is between 1A^4^ and 1B^4^-Cap^+^, and the lowest between 1B^4^-Cap^−^ and 1B^4^-Cap^+^. A “comparison of comparisons” was performed, whereby each of comparisons A, B, and C for Line 6 was equated to the Line 1 counterpart. Lists of shared and unique genes for comparisons A, B, and C were generated (supplementary table S6, Supplementary Material online). For comparison C, B^4^-Cap^−^ versus B^4^-Cap^+^, 26 genes are common between Line 6 and Line 1; nine of these are more highly expressed in Cap^−^ forms compared with the Cap^+^, and include four flagella genes and five genes of unknown function. The remaining 17 genes are more highly expressed in the Cap^+^ forms than in the Cap^−^, and include seven CAP genes, a transcriptional regulator, an inorganic ion transport gene and eight genes of unknown function. Together, the results corroborate the finding that capsules and flagella are mutually exclusive. A similar finding has recently been reported in *Cronobacter sakazakii*, in which induction of colanic acid biosynthesis is accompanied by a reduction in flagella gene expression (Chen et al. 2018).

### Genes Encoding Ribosomal Proteins Are Overexpressed in 6B^4^

The recently proposed ribosome–RsmAE model of 1B^4^ capsule switching postulates that capsulation is controlled by the combined intracellular pool of ribosomes, RNA-binding proteins RsmA/RsmE and *pflu3655–pflu3657* mRNA (Remigi et al. 2019, fig. 6*A* and *B*). According to the model, ribosomes and RsmAE compete for binding sites in *pflu3655–pflu3657* mRNA; ribosome binding results in translation of transcriptional activators Pflu3655, Pflu3656, and Pflu3657, the downstream targets of which include *pflu3655–pflu3657* and capsule biosynthetic genes. Thus, transcription of *pflu3655–pflu3657* activates a positive feedback loop that triggers capsulation. RsmAE binding results in inhibition of *pflu3655–pflu3657* mRNA translation, and thus promotes the noncapsulated state. RsmA and RsmE function (discussed in Vakulskas et al. 2015; Remigi et al. 2019) is under the immediate control of two small, noncoding RNAs: rsmY and rsmZ. These are each predicted to contain multiple RsmAE binding sites, allowing them to act as molecular sponges that remove RsmAE from the pool. Finally, rsmYZ expression is itself under the control of at least two master regulators: positive control by the GacA/GacS phosphorelay system (*pflu2189/pflu3777*; Lapouge et al. 2007) and repression by the transcriptional regulator MvaT (*pflu4939*; Brencic et al. 2009).

**Fig. 6.**
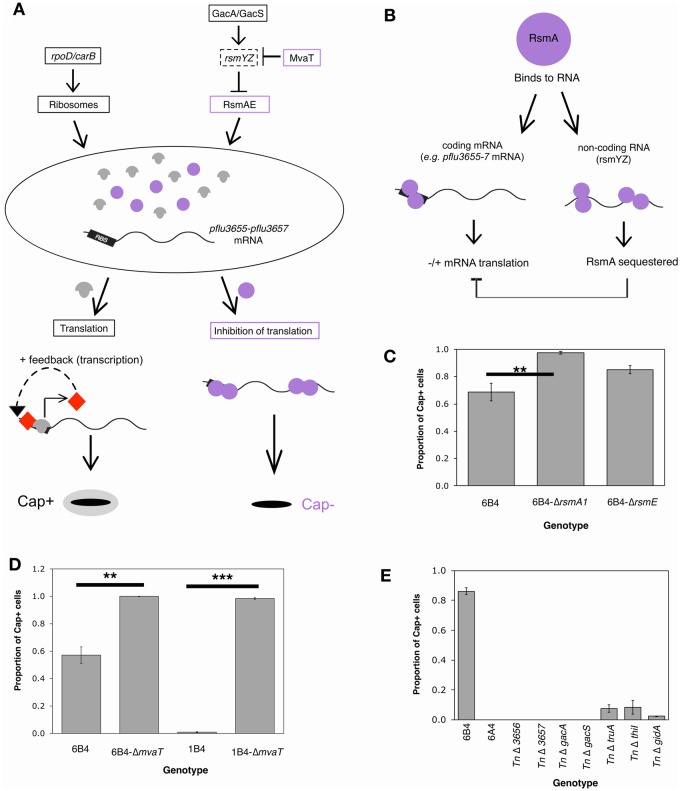
The ribosome–RsmAE model of capsule switching in 6B^4^. (*A*) As *per*Remigi et al. (2019), relative pools of three molecules determine Cap state: ribosomes (gray mushrooms), RsmAE (purple circles), *pflu3655* mRNA. Initially, ribosomes and RsmAE compete for binding sites on *pflu3655* mRNA. If ribosomes bind then translation follows, giving Pflu3655 (red diamond) and CAP synthesis. Pflu3655 forms a positive feedback loop (*pflu3655* transcription) that maintains the Cap^+^ state. Change to Cap^−^ requires RsmAE to outcompete the other components (reduction of *pflu3655* mRNA and/or production of RsmA/E). Intracellular components are predicted to influence Cap positively (black boxes) or negatively (purple boxes) by altering relative pools. Solid outlines = components with supporting evidence, dotted lines = untested. (*B*) Model of RsmAE function in *Pseudomonas fluorescens* SBW25. RsmAE binds to RNA sequences found in short RNAs (rsmYZ; right) and various promoters (left). The net binding of RsmAE to a promoter affects translation of the mRNA through competitive binding with other translational machinery (e.g., ribosomes). (*C*) *rsmA1* deletion in 6B^4^ increases Cap^+^ in exponential phase (two sample *t-*test *P* = 1.112 × 10^−3^). (*D*) *mvaT* (*pflu4939*) deletion in 6B^4^ or 1B^4^ increases Cap^+^ in exponential phase (Wilcoxon test *P* = 0.009761; two sample *t*-test *P =* 1.952 ×10^−15^). (*E*) Nonpolar insertions in *pflu3656*, *pflu3657*, RsmAE regulators (*gacA*, *gacS*), and translation machinery (*truA*, *gidA*, *thiI*) reduce 6B^4^ capsulation in exponential phase. Bars = mean of 5 (*B*, *C*) or 3 (*D*) replicates, error bars 1 SE.

The above model predicts that genotypes with increased capsulation (such as 6B^4^) contain higher levels of *pflu3655–3657* mRNA as a result of a net increase in ribosomes. Consistent with the model, *pflu3655*, *pflu3656*, and *pflu3657* mRNA levels are significantly higher in 6B^4^-Cap^+^ and 6B^4^-Cap^−^ than in 6A^4^. Indeed *pflu3655* is the most highly differentially expressed gene in all three RNA-seq comparisons: it is expressed 572-fold more highly in 6B^4^-Cap^+^ than in 6A^4^, 31-fold more highly in 6B^4^-Cap^−^ versus 6A^4^, and 19-fold more highly in 6B^4^-Cap^+^ versus 6B^4^-Cap^−^ (supplementary tables S3–S5, Supplementary Material online). Furthermore, the *rpoD* mutation leads to an increase in the mRNA of genes encoding ribosomal proteins (fig. 6*A*); 40 and 43 (of 53) genes encoding ribosomal protein show increased expression in 6B^4^-Cap^+^ compared with 6A^4^ and 6B^4^-Cap^−^, respectively (supplementary table S7, Supplementary Material online). Although only three of these show statistical significance (*rpmG*, *rpmB*, and *rpsT* are more highly expressed in 6B^4^-Cap^+^ than in 6A^4^; supplementary text S3, Supplementary Material online), there is a clear pattern of higher expression in the capsulated form. Further binomial tests provide strong evidence for increased expression of genes encoding ribosomal proteins in 6B^4^-Cap^+^ versus 6A^4^ and 6B^4^-Cap^−^ (*P <* 0.001; supplementary text S3, Supplementary Material online). The second major constituent of mature ribosomes is ribosomal RNA, encoded by the 23S, 16S, and 5S rRNA genes. The RNA-seq data does not provide quantifiable differences in the expression of rRNA genes. This is because rRNA is highly abundant in total RNA preparations (∼80% to 90% in exponential growth; Tissieres and Watson 1958), and so rRNA was removed from the preparation to quantify mRNA.

Taken together, the observed increases in *pflu3655–pflu3657* and ribosomal mRNAs are consistent with the *rpoD* mutation generating an increase in ribosome expression and thus increasing the probability of capsulation.

### Manipulating Components of the Model Generates Changes in 6B^4^ Capsule Switching

To test whether the ribosome–RsmAE model (fig. 6*A* and *B*) underpins switching in 6B^4^, components of the model were manipulated to bias the switch in favor of ribosomes (Cap^+^) or RsmA/RsmE (Cap^−^). The switch was tipped in favor of ribosomes by decreasing RsmA/E activity in two ways. First, *rsmA1* (*pflu4746*) and *rsmE* (*pflu4165*) were individually deleted from 6B^4^, giving 6B^4^-Δ*rsmA1* and 6B^4^-Δ*rsmE* (supplementary text S2, Supplementary Material online). A capsule counting assay revealed increases in capsulation (fig. 6*C*); in particular, deletion of *rsmA1* resulted in significantly higher levels of capsulation (one-sided *t*-test *P* = 0.001112), bringing the percentage of capsulated cells to almost 100%. The difference in capsulation between 6B^4^-Δ*rsmA1* and 6B^4^-Δ*rsmE* may be partially explained by different expression profiles: in *P. fluorescens* CHA0, RsmA expression has been shown to be relatively constant, whereas RsmE is expressed at very low levels during exponential growth (Reimmann et al. 2005).

Second, the model predicts RsmAE activity to be reduced by deletion of *mvaT*, which encodes a transcriptional repressor of *rsmZ*—itself a negative regulator of RsmAE—in *Pseudomonas aeruginosa* (Brencic et al. 2009). Deletion of *mvaT* (*pflu4939*) from 6B^4^ or 1B^4^ results in a significant increase in capsulation (*P* < 0.01; fig. 6*D*, supplementary text S2, Supplementary Material online).

In contrast to the above, any bias of the switch machinery in favor of RsmAE is expected to inhibit translation of *pflu3655–pflu3657* mRNA, and thus reduce capsulation. Support for this side of the model comes from the transposon mutagenesis screen (supplementary table S1, Supplementary Material online). First, three capsule-reducing insertions were obtained in the GacA/GacS two-component sensory system, which is a negative regulator of RsmAE in γ-proteobacteria (Lapouge et al. 2007, fig. 6*A*). Inactivation of these genes is expected to increase RsmAE expression and decrease capsulation. Indeed, 6B^4^-TnCre-*gacA* and 6B^4^-TnCre-*gacS* (Cre-deleted forms of the transposon mutants, see “Materials and Methods” section and supplementary text S2, Supplementary Material online) showed a complete absence of capsulation (fig. 6*E*). Second, four transposon insertions were identified in genes involved in the production of mature tRNAs: two in *gidA/mnmG* (*pflu6129*), one in *truA* (*pflu4189*), and one in *thiI* (*pflu0349*; reviewed in Yacoubi et al. 2012). Each of these insertions resulted in a reduction in capsulation (fig. 6*E*). Although not lethal, disruption of each tRNA modification gene is expected to reduce translational speed (Yacoubi et al. 2012; reviewed in Shepherd and Ibba 2015), suggesting a role for efficient translation in capsulation.

The ability to increase and decrease 6B^4^ capsules by manipulating components of the 1B^4^ ribosome–RsmAE circuitry (as predicted by the model) demonstrates that the same intracellular architecture underpins switching in both genotypes.

## Discussion

In this work 6B^4^ has been extensively characterized. Its phenotype and genotype have been compared with those previously reported for 1B^4^—a strain evolved in parallel to, but independently of, 6B^4^ (Beaumont et al. 2009; Gallie et al. 2015). 6B^4^ and 1B^4^ populations show elevated levels of CAP-based capsule expression and emergent colony switching (figs. 1*B*–*E* and 2*A*). The phenotype is realized by two distinct genetic routes, culminating in a mutation in either *rpoD* (Line 6) or *carB* (Line 1). Both mutations promote increased expression of mRNA encoding positive regulators of the CAP biosynthetic machinery. These regulators also activate their own transcription, forming a positive feedback loop that results in bistable capsule expression (outlined in Remigi et al. 2019, fig. 6*A* and *B*).

Line 6 and Line 1 were derived from a single clonal ancestor (*P. fluorescens* SBW25). This means that the genotypes of interest, 6B^4^ and 1B^4^, share an evolutionary history of many millions of years followed by a comparatively minuscule period of several weeks of independent evolution in experimental microcosms. Given the extensive shared history, it is not surprising that the same phenotype emerged in both lineages. It is surprising, however, that such different genetic routes generate the same phenotype.

Repeated phenotypic evolution has been documented many times in both the laboratory (Riehle et al. 2001; Cooper et al. 2003; Fong et al. 2005; Ostrowski et al. 2005; Bantinaki et al. 2007; Meyer et al. 2012; Lindsey et al. 2013) and natural populations (Nachman et al. 2003; Rosenblum et al. 2004; Stern and Frankel 2013; Riveron et al. 2014). In many of these examples, repeated phenotype evolution is determined by changes in the same gene or molecular pathway (Meyer et al. 2012; Lindsey et al. 2013; Riveron et al. 2014; Bertels et al., 2018). The fact that colony switching in Line 6 and Line 1 arise by different genetic pathways—despite extreme shared ancestry—is surprising. At first glance, *rpoD* and *carB* seem functionally unrelated and, as such, it is natural to assign them to separate functional compartments. However, this work shows that the two genes are connected at the level of their effects on the ribosome–RsmAE pool: both mutations increase expression of ribosomal genes (supplementary table S7, Supplementary Material online; Gallie et al. 2015; Remigi et al. 2019) tipping the switch in favor of CAP mRNA translation (fig. 6*A* and *B*).

The precise molecular mechanisms by which *rpoD* and *carB* mutations alter ribosomal gene expression remain to be elucidated. However, it is conceivable that the *rpoD* mutation directly increases the transcription of one or more ribosomal genes. A point mutation in *Salmonella typhimurium rpoD* has recently been shown to increase transcription from *rpsT* (Knöppel et al. 2016). In the case of the *carB* mutation, which perturbs intracellular pyrimidine pools (Gallie et al. 2015), the reported influence of nucleotide triphosphate concentrations on *rrn* promoters may play a mechanistic role (Gaal et al. 1997; Schneider et al. 2002, 2003; Murray et al. 2003; Schneider and Gourse 2003). If cellular components show a high degree of connectivity, it follows that many other factors could also affect the switch circuitry. Possible candidates include those affecting capsule expression and identified via the transposon mutagenesis screens (e.g., *hslO*, *sahA*, *ndk*; supplementary table S1, Supplementary Material online; Gallie et al. 2015).

In stark contrast to the disparate molecular evolution of 6B^4^ and 1B^4^, repeated bouts of evolution from the same immediate ancestor of the 6B^4^ switching genotype, namely, 6A^4^, resulted in re-evolution of the switching genotype by mutations solely in *rpoD* (fig. 4*A*). Similar repeated bouts of evolution from 1A^4^ (the immediate ancestor of the Line 1 switching genotype) resulted in switching types with mutations in genes encoding the determinants of pyrimidine biosynthesis (five in *carB*, one in *pyrH*; Gallie et al. 2015). In other words, the comparatively tiny portion of evolutionary history for which Line 6 and Line 1 diverged—several weeks compared with millions of years of common history—has a significant impact on molecular evolution.

The distinct classes of switcher mutations in Line 6 and Line 1 result from positive epistatic interactions: whereas both types of switch-causing mutations presumably arise in both backgrounds, *rpoD* mutations outcompete *carB* mutations in 6A^4^, and vice versa in 1A^4^ (fig. 5*C*). Precisely which of the preswitcher mutations in each evolutionary series contribute to the observed epistatic effects remains to be tested. In the case of Line 6, the two *nlpD* mutations immediately preceding the *rpoD* mutation are prime candidates for two reasons. First, *nlpD* is the only locus that is mutated in Line 6 but not Line 1 (figs. 1*B* and 2*A*). Second, *nlpD* is immediately upstream of *rpoS* (*pflu1302*), which encodes the stationary phase sigma factor RpoS (σ^38^). RpoS and RpoD (together with other sigma factors) compete for binding of core RNA polymerase (Ishihama 2000; Mauri and Klumpp 2014), and so their relative intracellular concentration affects the expression level of their respective regulons (Gross et al. 1998; Mauri and Klumpp 2014). It is possible that the *nlpD* mutations, in addition to altering colony morphology via a reduction of NlpD/AmiC activity, also alter the expression of *rpoS*. Indeed, a promoter for *rpoS* has previously been reported within *E. coli* and *P. aeruginosa nlpD* (Lange and Hengge-Aronis 1994; Takayanagi et al. 1994; Kojic and Venturi 2001). A change in RpoS concentration could conceivably set the stage for compensatory mutations in RpoD.

Understanding the molecular bases of adaptive phenotypes continues to present significant challenges even when aided by high-throughput genomic technologies. As shown here and elsewhere (Larsen et al. 2008; Bershtein et al. 2015; Gallie et al. 2015; Grenga et al. 2017; Carvalho et al. 2018), mutations—particularly those in central metabolism—can have complex effects that extend well beyond the immediate neighborhood of gene function. The point mutations in two seemingly unrelated genes (*rpoD* and *carB*) can generate stochastic capsule switching draws attention to the interconnectedness of cell physiology and highlights the extensive mutational opportunities available to evolution.

## Materials and Methods

### Bacterial Strains, Plasmids, and Media

Details of bacterial strains and plasmids used are provided in supplementary text S2, Supplementary Material online. Unless otherwise stated, *P. fluorescens* strains were grown for 24 h at 28 °C in shaken microcosms with 6 ml King’s Medium B (KB; Ward et al. 1954). Strains were assayed for cellulose production by overnight growth on KB agar containing 200 μg ml^−1^ calcofluor (Fluorescent Brightener 28). Where indicated, 2 mM uracil (Sigma-Aldrich, U1128) was added to KB agar. Antibiotics were used at the following concentrations: tetracycline (12.5 μg ml^−1^; Tc); kanamycin (100 μg ml^−1^; Km); nitrofurantoin (100 μg ml^−1^; NF); d-cycloserine (800 μg ml^−1^).

### Microscopy

Cell microscopy was performed using a Zeiss Axiostar Plus bright field microscope, coupled with fluorescence lighting for calcofluor visualization. Microscopy images were cropped and processed in Preview as indicated in figure legends.

### Capsule Counting Assay

Capsule staining and the counting assay were performed as previously described in Gallie et al. (2015). Briefly, for each strain to be assayed, three to five single colonies were grown to stationary phase in KB cultures. Cultures were transferred to fresh KB and grown to mid-exponential or early stationary phase as indicated. Cells were stained with 1:8 diluted India ink (Pébéo) and photographed under bright field 60× magnification. Capsule expression was recorded manually for 500 cells *per* replicate (≤100 cells *per* photograph).

### Gene Deletions and Mutation Construction

Gene deletions were constructed by pUIC3-mediated two step allelic exchange as described elsewhere (Zhang and Rainey 2007). For details of genetic constructs supplementary text S2; see Supplementary Material online for details.

### Transposon Mutagenesis

6B^4^ was subjected to random mutagenesis as described in Giddens et al. (2007). Approximately 10,000 transposon mutants from 11 independent conjugations were screened on LB+Km agar plates, on which 6B^4^ mutants typically form opaque colonies after ∼72 h. Translucent or otherwise different colonies were selected and screened microscopically for obvious alterations in capsule expression. Mutants of interest were purified and insertion sites determined by AP-PCR. In selected strains, the bulk of the transposon was deleted leaving 189 bp at the insertion site (“TnCre-”genotypes) and eliminating polar effects.

### Isolation and Analysis of EPS

EPS was isolated and processed from 6A^4^ and 6B^4^ in parallel with that from SBW25, 1A^4^, and 1B^4^ previously reported in Gallie et al. (2015). EPS analysis was performed by The Callaghan Research Institute (New Zealand).

### Genome Sequencing of 6B^4^

A colony of 6B^4^ was grown in a microcosm. Cap^+^ and Cap^−^ fractions were separated by centrifugation and genomic DNA isolated from each fraction using the cetyl trimethyl ammonium bromide (CTAB) method. Equal quantities of Cap^+/^^−^ DNA were mixed, and whole genome resequencing was performed (Illumina; Massey University, New Zealand). Point mutations were identified by aligning ∼4.8 million 36 bp reads to the SBW25 genome (Silby et al. 2009) via SOAP2 (Li et al. 2009). Reads with more than two mismatches and/or that could not be uniquely aligned to the genome were discarded. A total of 4,028,678 reads aligned, giving a mean coverage of 21.57. 98.8% of the genome was covered by at least four nucleotides. Within this 98.8%, all positions with a minimum variation frequency of 0.8 were considered candidate point mutations; candidate insertions and deletions were identified by analyzing genomic regions with unusual coverage and BLAST analyses of discarded sequences. All candidate loci were checked by PCR amplification and Sanger sequencing from 6B^4^ and, where confirmed, were chronologically ordered by Sanger sequencing across Line 6. Genome sequence and analysis files are available on request.

### Re-Evolution of Switchers from 6A^4^

Nine independent switcher genotypes were isolated from 6A^4^ in static microcosms, according to the REE protocol (Beaumont et al. 2009). Each switcher was purified, and the *rpoD* gene sequenced. Three strains, one carrying each of the *rpoD* mutations (6B^4^, Re1, Re9), were checked for bi-directional capsule switching (supplementary text S1, Supplementary Material online).

### Growth Curves and Analysis

Eight colonies *per* strain were grown independently in 200 μl KB (26 °C, 200 rpm). Two microliters of each were grown in 198 μl fresh KB at 26 °C (BioTek Epoch 2 plate reader; OD_600_ measured at 5 min intervals, 5 s of 3 mm orbital shaking preceding each read). Mean and SE of all wells *per* strain were used to draw figure 4*D*. *V*_max_ (maximum growth rate) and lag time were calculated using a sliding window of six time points during exponential growth (between 1 and 24 h, based on observation of growth curves) using Gen5 Software version 3.00.19.

### RNA-seq Analysis

For each of 6A^4^ and 6B^4^, three single colonies were grown in KB, diluted 1:1,000 into 20 ml KB in 250 ml flasks and grown to mid-exponential phase (∼OD_600_ of 0.4–0.6). Total RNA was harvested; for 6A^4^, 0.5 ml of culture were pelleted and resuspended in 1 ml of RNAlater (Ambion). For 6B^4^, Cap^−^ cells were harvested from larger culture volumes by centrifugation and resuspended in RNAlater to achieve a similar cell density to that of the Cap^+^ aliquot. All mRNA extractions proceeded using a RiboPure Bacteria Kit (Ambion). Specific depletion of rRNA (Ribo-Zero rRNA Removal Kit [Bacteria], Illumina), normalized mRNA-seq library preparation (TruSeq Stranded total RNA kit, Illumina) and 100 bp paired-end Illumina HiSeq 2500 sequencing was performed by New Zealand Genomics Limited (University of Otago, New Zealand; GEO submission number GSE116490). The data were analyzed with Bowtie2 (Langmead and Salzberg 2012), HTSeq (Anders et al. 2015), and R package DESeq2 (Love et al. 2014). First, RNA-seq data sets were mapped to the SBW25 genome (annotation file: GenBank NC_012660) via Bowtie2 with default settings. The coverage *per* gene was determined with HTSeq. Differentially expressed genes were identified by applying DESeq2. The standard workflow in https://bioconductor.org/packages/release/bioc/manuals/DESeq2/man/DESeq2.pdf (last accessed 8 March 2019) was used, except that the alpha parameter was set to 0.3 to reduce the number of genes falsely classified as not significantly differentially expressed. Three comparisons were made: 6A^4^ versus 6B^4^-Cap^−^, 6A^4^ versus 6B^4^-Cap^+^, and 6B^4^-Cap^−^ versus 6B^4^-Cap^+^ (supplementary tables S3–S5, Supplementary Material online). The corresponding comparisons for Line 1 are available elsewhere (Gallie et al. 2015).

### Fitness Assays

Four (Line 6) or eight (Line 1) single colonies of each competitor were grown independently in shaken KB (28 °C). Four (Line 6) or eight (Line 1) competition microcosms were inoculated with ∼5 × 10^6^ cells of each competitor and incubated statically at 28 °C for 72 h. Competitor frequencies were determined by plating on KB agar at 0 and 72 h. Competing genotypes were readily distinguished by their distinctive morphologies and differing response to uracil (adding 2 mM uracil to KB agar visibly reduces switching caused by *carB* mutations, while having no effect on *rpoD* mutant switching). Relative fitness was expressed as the ratio of Malthusian parameters (Lenski et al. 1991). Deviation of relative fitness from 1 was determined by one-sample *t*-tests.

### Statistical Analyses

To detect differences in capsulation levels or nucleotide concentrations between two strains, two-sample *t-*tests (parametric or Welch) or, where normality assumptions were violated, Wilcoxon rank sum tests were applied. Kruskal–Wallis tests were used to detect differences in capsulation levels across the three *rpoD* mutant strains. Exact binomial tests were used to detect differences in ribosomal gene expression between morphotypes in the RNA-seq data (see also supplementary text S3, Supplementary Material online). All analyses were performed in R version 3.3.3. On graphs: *0.05 < *P <* 0.01, **0.01 < *P <* 0.001, ***P < 0.001.

## Supplementary Material

Supplementary Data
