# Supplementary material for: Repeated Phenotypic Evolution by Different Genetic Routes in Pseudomonas fluorescens SBW25
Source: Mol Biol Evol. 2019 Mar 5;36(5):1071–85. doi: 10.1093/molbev/msz040 (PMC6519391; doi:10.1093/molbev/msz040)
Supplement: Supplementary Data [file msz040_supp.zip › supplementary_information.docx]

**SUPPLEMENTARY INFORMATION**

**Repeated phenotypic evolution by different genetic routes in *Pseudomonas fluorescens***

*Jenna Gallie, Frederic Bertels, Philippe Remigi, Gayle C Ferguson, Sylke Nestmann, Paul B Rainey*

The supplementary information contains the following:

Supplementary Texts S1-S3 (pages 2-20)

Supplementary Figure S1 (page 21)

Supplementary Table S1-S7 (legends for accompanying excel files; pages 22-23)

Supplementary References (page 24)

**Supplementary Text S1
Characterization of capsulation in switcher genotypes**

**1.1 Capsule size in 6B^4^ versus 1B^4^**

In order to investigate relative capsule size in 6B^4^ versus 1B^4^, each strain was grown from single colonies (KB agar, 28˚C, 48 hours) through pre-cultures (KB, 28˚C, 200 rpm, 24 hours) to late-exponential phase (KB, 28˚C, 200 rpm; ~6 hours). Cells from each culture were stained with India ink (1:8 dilution) and photographed under the bright field microscope (x60 magnification). Ten cells were chosen from each image. For each of the chosen cells, four measurements were taken (**Table 1.1**; note that the measurement units were mm on a computer screen. This means they only make sense in relation to each other, rather than as absolute measurements):

1. Length of the cell plus capsule
2. Width of the cell plus capsule
3. Length of the cell inside the capsule
4. Width of the cell inside the capsule

Next, using the formula for area of an oval (area=r *x* r *x* π), two areas were calculated for each measured cell (**Table 1.1**):

1. The area of the capsule+cell
2. The area of the cell

Area (ii) was subtracted from area (i) to give area of the capsule (**Table 1.1**). Next, a test was run to check for a difference in cell size between 1B^4^ and 6B^4^. No evidence was found (two sample *t*-test *-p*=0.5326). Then, the capsule areas were used to perform a two sample *t*-test to check for a difference in area. The mean area of the 6B^4^ capsule is 165.8 units^2^, and that of 1B^4^ capsule is 65.19 units^2^. These are significantly different (*t-*test *p*=9.602x10^-10^). The 95% CI for the true difference in means is 82.30 – 118.99 units^2^. This equates to the 6B^4^ capsule area being between 1.26 (82.30/65.19) and 1.83 (118.99/65.19) times that of the 1B^4^ capsule.

| **Str.** | **No.** | **Capsule+cell** | | **Cell** | | **Area** | | |
| --- | --- | --- | --- | --- | --- | --- | --- | --- |
|  |  | **Length** | **Width** | **Length** | **Width** | **Caps+cell** | **cell** | **Caps** |
| 6B^4^ | 1 | 16 | 14 | 7 | 1.5 | =(0.5*16)x(0.5*14)* π= 175.93 | =(0.5*7)x(0.5*1.5)* π= 8.25 | =175.93-8.25=167.68 |
|  | 2 | 17 | 14 | 8 | 1 | 186.92 | 6.28 | 180.64 |
|  | 3 | 17 | 13 | 6 | 1 | 173.57 | 4.71 | 168.86 |
|  | 4 | 14 | 11 | 4 | 1 | 120.95 | 3.14 | 117.81 |
|  | 5 | 17 | 14 | 6 | 1 | 186.92 | 4.71 | 182.21 |
|  | 6 | 17 | 15 | 7 | 1 | 200.28 | 5.50 | 194.78 |
|  | 7 | 18 | 14 | 8 | 1 | 197.92 | 6.29 | 191.64 |
|  | 8 | 16 | 13 | 6 | 1 | 163.36 | 4.71 | 158.65 |
|  | 9 | 16 | 13 | 6 | 1 | 163.36 | 4.71 | 158.65 |
|  | 10 | 15 | 12 | 5 | 1 | 141.37 | 3.93 | 137.44 |
| 1B^4^ | 1 | 12 | 8 | 7 | 1 | 75.40 | 5.50 | 69.90 |
|  | 2 | 14 | 7 | 7 | 1 | 76.97 | 5.50 | 71.47 |
|  | 3 | 10 | 6 | 5 | 1 | 47.12 | 3.93 | 43.20 |
|  | 4 | 12 | 6 | 8 | 1 | 56.55 | 6.28 | 50.27 |
|  | 5 | 13 | 7 | 8 | 1 | 71.47 | 6.28 | 65.19 |
|  | 6 | 11 | 8 | 6 | 1 | 69.12 | 4.71 | 64.40 |
|  | 7 | 14 | 8 | 7 | 1 | 87.96 | 5.50 | 82.47 |
|  | 8 | 12 | 9 | 6 | 1 | 84.82 | 4.71 | 80.11 |
|  | 9 | 12 | 6 | 9 | 1 | 56.55 | 7.07 | 49.48 |
|  | 10 | 13 | 8 | 8 | 1 | 81.68 | 6.28 | 75.40 |

**Table 1.1: Measurements for capsules and cells of 6B^4^ and 1B^4^.**

**1.2 Demonstration of bi-directional ON/OFF capsule switching in *rpoD* mutants**

*Isolation of 15 potential switcher genotypes from 6A^4^*

In an attempt to re-evolve independent switcher genotypes from 6A^4^ (the immediate ancestor of the Line 6 switcher, 6B^4^), 56 independent KB microcosms were each founded with 6A^4^ and allowed to evolve in a static environment before plating. From this process, a total of 15 potential switcher genotypes were isolated from 15 independent microcosms. Each of these genotypes produced colonies distinct from 6A^4^ colonies, and carried mutations 3-8 of the Line 6 mutational series plus at least one independent, final mutation (identified either by targeted sequencing of *rpoD*, or, where no *rpoD* mutation was found, NGS Illumina; see **Table 1.2** below). Following a series of phenotypic tests outlined in the following sections, nine independently evolved CAP switcher genotypes were identified from amongst these 15 candidate genotypes.

| **Original name** | **Details of final mutation(s)** | | | | **Switcher?** | **Final name (if switcher)^2^** |
| --- | --- | --- | --- | --- | --- | --- |
|  | ***pflu*^1^** | **Gene name** | **Nucleotide change** | **Amino acid change** |  |  |
| 6B^4^ | 5592 | *rpoD* | t1682c | V561A | Yes | 6B^4^ |
| JG58 | 4939 | *pflu4939* | c91t | Q31STOP | No | - |
|  | 5720 | *sahA* | t1097a | I366N |  |  |
| JG59 | 5592 | *rpoD* | a1723c | T575P | Yes | Re1 |
| JG60 | 3427 | *pflu3427* | g1561a | A521T | No | - |
|  | 4939 | *pflu4939* | a260c | N87T |  |  |
| JG61 | 4939 | *pflu4939* | g332a | W111STOP | No | - |
| JG62 | 5592 | *rpoD* | a1723c | T575P | Yes | Re2 |
| JG63 | 5592 | *rpoD* | a1723c | T575P | Yes | Re3 |
| JG64 | 4939 | *pflu4939* | g321a | W107STOP | No | - |
| JG65 | 5592 | *rpoD* | a1723c | T575P | Yes | Re4 |
| JG66 | 5592 | *rpoD* | a1723c | T575P | Yes | Re5 |
| JG67 | 5592 | *rpoD* | a1723c | T575P | Yes | Re6 |
| JG68 | 5592 | *rpoD* | a1723c | T575P | Yes | Re7 |
| JG69 | 1225 | *wspR* | c583t | R195C | No | - |
| JG70 | 1225 | *wspR* | c583t | R195C | No | - |
| JG71 | 5592 | *rpoD* | a1745c | Q582P | Yes | Re8 |
| JG72 | 5592 | *rpoD* | a1723c | T575P | Yes | Re9 |

**Table 1.2:** **Mutations in 15 potential switcher genotypes isolated from 6A^4^.** ^1^JG58 and JG60 each carry two mutations additional to 6A^4^. It is unknown which of the two mutations causes the colony phenotype change.

*Colony and cell phenotypes of the 15 genotypes*

Each of the 15 genotypes was streaked onto KB agar and grown at 26˚C for 68 hours. The Colony photographs below were taken with a Leica MS5 dissection microscope and a VWR Visicam 1.3. All colony photographs were taken under the same magnification (x1.6); brightness and contrast of some photographs altered in Microsoft word.


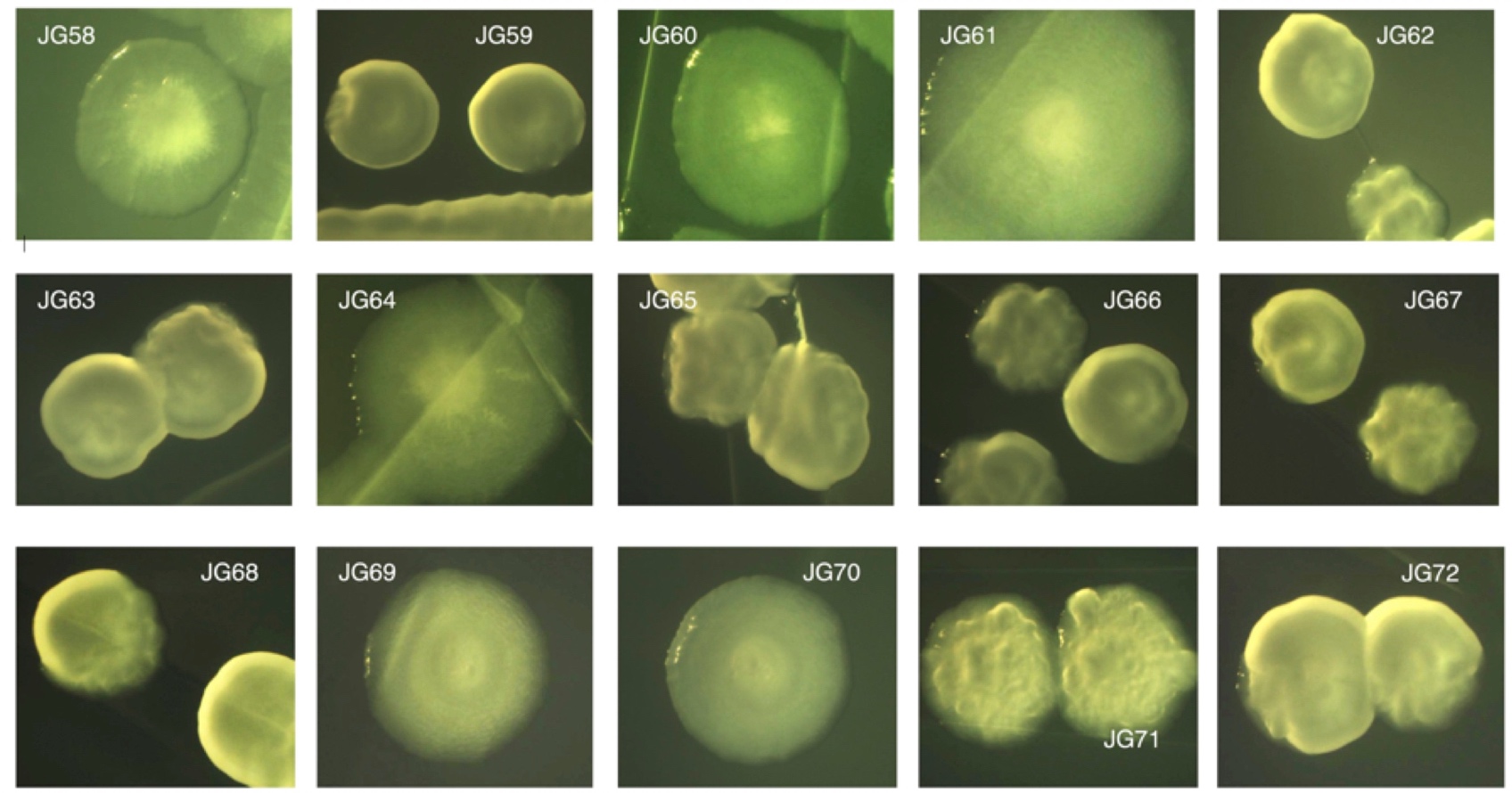


Next, colonies were streaked onto KB agar plates, incubated at 26˚C for 48 hours before transfer of a single colony to 4 ml KB cultures. These were incubated at 26˚C, 200 rpm for 48 hours before staining with India ink and viewing with a VWR Visiscope bright field microscope. The contrast and brightness of some images altered in Microsoft Word.


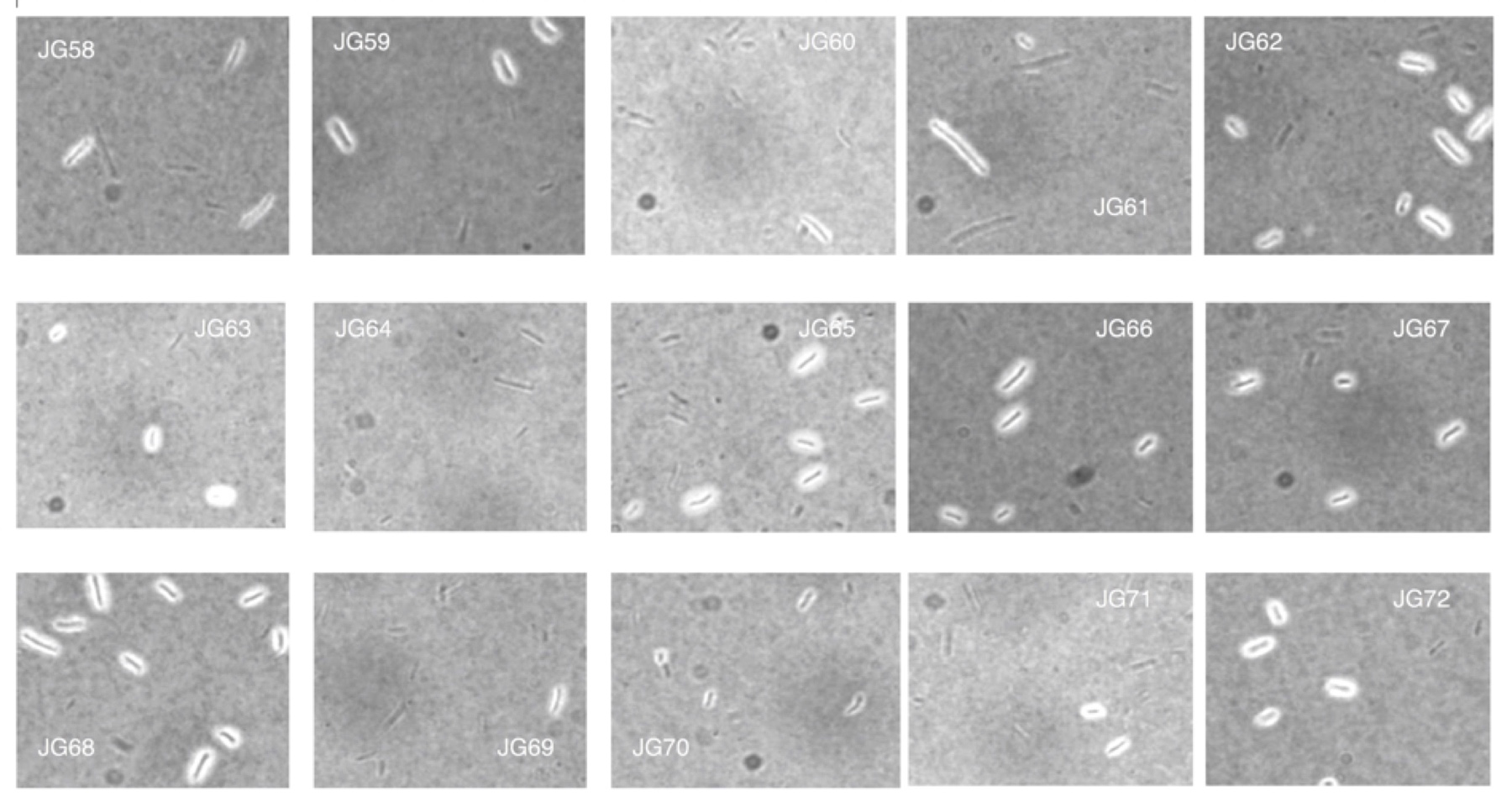


From the above colony and cell assays, we concluded that genotypes JG58, JG60, JG61, JG64, JG69 and JG70 were unlikely to be true switcher types. This was because these genotypes did not give rise to two distinct colony types; instead they generated one, uniform (and wild type-like) colony phenotype. These genotypes were those containing mutations in *pflu4939* or *wspR*. Because of their colony phenotype they were discarded from the analysis process, leaving only the nine genotypes with *rpoD* mutations as capsule switching contenders.

*Growth of Cap^+^/Cap^-^ mixed populations from single cells in a 96 well plate*

The point of this assay was to test whether the genotypes of interest are capable of generating a mixed Cap^+^/Cap^-^ population from both single Cap^+^ and single Cap^-^ cells. In other words, it tests whether capsule switching is (a) unidirectional, or (b) bidirectional. If some populations founded by single cells of either type contain cells of only one type, this would support hypothesis (a). Alternatively, hypothesis (b) would be supported if all populations contain a mixture of cell types. The assay was performed with a representative genotype of each of the three different *rpoD* mutations: 6B^4^ (*rpoD* t1682c), JG59 (Re1; *rpoD* a1723c) and JG71 (Re9; *rpoD* a1745c).

6B^4^, JG59 and JG71 cells were streaked from freezer stocks onto KB agar. After 48 hours growth at 26˚C, a single colony from each genotype was used to inoculate a KB culture. The colony chosen was opaque, as prior experience suggests that, following overnight incubation in a shaking microcosm, the population would contain approximately equal proportions of non-capsulated and capsulated cells. The inoculated microcosms were incubated for 16 hours (at 26˚C, 200 rpm) prior to thorough vortexing and microscopic examination to ensure complete separation of cells. Then, a capsule counting assay was performed to determine the starting proportions of Cap^+^ and Cap^-^ cells in each culture (mean ± standard error of five samples from each culture; **Table 1.3**):

| **Genotype** | **Proportion of cells capsulated in founding culture (raw data)** | **Mean** | **SE** |
| --- | --- | --- | --- |
| 6B^4^ | 0.144, 0.098, 0.136, 0.122, 0.408 | 0.1816 | 0.057136328 |
| JG59 | 0.744, 0.664, 0.62, 0.74, 0.682 | 0.69 | 0.0235117 |
| JG71 | 0.14, 0.172, 0.138, 0.23, 0.136 | 0.1632 | 0.04016466 |

**Table 1.3: Proportion of capsulated cells in populations of the three *rpoD* switchers.** Proportions were determined in each of five independent KB microcosms (founded from a single colony) during early stationary phase.

For each of the three genotypes, 95 μl of fresh KB was measured into the wells of a 96 well plate. A sample of the appropriate overnight culture was diluted by a factor of 7 in Ringers Solution, and 5 μl of this dilution was used to inoculate each well. On the basis that a 16-hour overnight KB culture contains approximately 10^9^ cells *per* ml, each well was predicted to receive an average of 0.5 cells. After 24 hours’ growth, the wells showing growth vs no growth was recorded for each plate, and the mixed Cap^+^/Cap^-^ composition of each “growth” well was checked by India ink staining and microscopy. Then, assuming a Poisson distribution, the number of “no growth” wells was used to calculate the number of wells theoretically inoculated by 1, 2, 3, and 4+ cells in each plate. Finally, using the starting proportion of Cap^+^ and Cap^-^ cells in the Table above, probability that the number of “growth” wells theoretically founded by 1 cell contained populations started by both single Cap^+^ and single Cap^-^ cells was calculated. If this proportion was >0.95, we state with 95% confidence that the 96 well plate contained at least one mixed Cap^+^/Cap^-^ population founded by a single Cap^+^ cell, *and* at least one mixed Cap^+^/Cap^-^ population founded by a single Cap^-^ cell. In other words, genotypes with a final probability of >0.9 were assumed to be bidirectional Cap^+^/Cap^-^ switchers.

*96 well plate for* *6B^4^*

The 96 well plate for 6B^4^ showed 43 wells with growth (shaded grey below), and 53 wells with no growth (unshaded). India ink staining followed by bright field microscopy showed a good mix of Cap+ and Cap- cells in each well that grew:

|  | 1 | 2 | 3 | 4 | 5 | 6 | 7 | 8 | 9 | 10 | 11 | 12 |
| --- | --- | --- | --- | --- | --- | --- | --- | --- | --- | --- | --- | --- |
| A |  |  |  |  |  |  |  |  |  |  |  |  |
| B |  |  |  |  |  |  |  |  |  |  |  |  |
| C |  |  |  |  |  |  |  |  |  |  |  |  |
| D |  |  |  |  |  |  |  |  |  |  |  |  |
| E |  |  |  |  |  |  |  |  |  |  |  |  |
| F |  |  |  |  |  |  |  |  |  |  |  |  |
| G |  |  |  |  |  |  |  |  |  |  |  |  |
| H |  |  |  |  |  |  |  |  |  |  |  |  |

It is known that there are 53 wells that were “inoculated” with 0 cells (no growth). Thus, we can use the Poisson distribution to calculate *λ,* the expected number of cells per well (*P*=probability of *y* cells founding any given well, *y*=number of cells of interest, *λ*=the expected number of cells *per* well, *e*=base of the natural logarithm):

$P\left( y \right)=\frac{\lambda^{y} x e^{-\lambda}}{y!}$

$\frac{53}{96}=e^{-\lambda}$

$\lambda= -(\ln\left( \frac{53}{96} \right))$ = 0.59405627797

This value of lambda was used to work out the theoretical occurrence of each number of cells inoculating a well (**Table 1.4**):

| **Number of cells** | **Actual or theoretical occurrence probability^a^** | **Number of wells^b^** | |
| --- | --- | --- | --- |
|  |  | **1 d.p.^c^** | **Rounded^d^** |
| 0 | 53/96=0.5520833333 | (53) | 53 |
| 1 | 0.3296857011 | 31.5 | 32 |
| 2 | 0.09741589402 | 9.4 | 9 |
| 3 | 0.01929017447 | 1.9 | 2 |
| 4+ | 0.00152489741 | 0.1 | 0 |
| Totals | 1.00 | 95.9 | 96 |

**Table 1.4: Poisson probabilities of population foundation by increasing numbers of 6B^4^ cells, and corresponding expected numbers of each in 96 independent populations. ^a^**Probability of the specified number of cells founding any given population. ^b^Corresponding expected number of 96 independent populations founded by specified number of cells to 1 decimal place (giving a total of 95.9 wells), and ^d^rounded to the nearest number of wells (96 wells).

The starting inoculum contained roughly a 0.1816 proportion of Cap^+^ cells (90.8/500). Thus, the probability that all 32 cells that were lone founders were capsulated (Pcap) is 1.96x10^-24^, while the probability that all 1 cell populations were founded by non-capsulated cells (Pnoncap) is 0.0016. Then, the probability that each cell type founded at least one population (Pboth) is 0.9984. These results are consistent with 6B^4^ cells being capable of switching in both directions.

$Pcap=\left( {0.1816}^{32} \right)=1.96x{10}^{-24}$

$$Pnoncap=\left( {0.8184}^{32} \right)=0.0016$$

$P\left( both \right)= 1-\left( Pcap+ Pnoncap \right)=0.9984$

*96 well plate for JG59 (Re1)*

This plate was not well sealed; 42 wells dried out and were discarded from the analysis (black shading). Of the remaining 54 wells, 11 showed growth (shaded grey) and 45 showed no growth (unshaded). India ink staining followed by bright field microscopy showed a good mix of Cap^+^ and Cap^-^ cells in each well that grew:

|  | 1 | 2 | 3 | 4 | 5 | 6 | 7 | 8 | 9 | 10 | 11 | 12 |
| --- | --- | --- | --- | --- | --- | --- | --- | --- | --- | --- | --- | --- |
| A |  |  |  |  |  |  |  |  |  |  |  |  |
| B |  |  |  |  |  |  |  |  |  |  |  |  |
| C |  |  |  |  |  |  |  |  |  |  |  |  |
| D |  |  |  |  |  |  |  |  |  |  |  |  |
| E |  |  |  |  |  |  |  |  |  |  |  |  |
| F |  |  |  |  |  |  |  |  |  |  |  |  |
| G |  |  |  |  |  |  |  |  |  |  |  |  |
| H |  |  |  |  |  |  |  |  |  |  |  |  |

It is known that there are 45 wells that were “inoculated” with 0 cells (no growth). Thus, we can use the Poisson distribution to calculate *λ,* the expected number of cells per well (*P*=probability of *y* cells founding any given well, *y*=number of cells of interest, *λ*=the expected number of cells *per* well, *e*=base of the natural logarithm):

$\frac{45}{54}=e^{-\lambda}$

$\lambda= -(\ln\left( \frac{45}{54} \right))$ = 0.18232155679

This value of lambda was used to work out the theoretical occurrence of each number of cells inoculating a well (**Table 1.5**):

| **Number of cells** | **Actual or theoretical occurrence probability^a^** | **Number of wells^b^** | |
| --- | --- | --- | --- |
|  |  | **1 d.p.^c^** | **Rounded^d^** |
| 0 | 45/54=0.833333333 | (45) | 45 |
| 1 | 0.15193463005 | 8.2 | 8 |
| 2 | 0.0138504793 | 0.7 | 1 |
| 3 | 0.00084174697 | 0.0 | 0 |
| 4+ | 3.98x10^-5^ | 0.0 | 0 |
| Totals | 1.00 | 53.9 | 54 |

**Table 1.5: Poisson probabilities of population foundation by increasing numbers of JG59 (Re1) cells, and corresponding expected numbers of each in 96 independent populations. ^a^**Probability of the specified number of cells founding any given population. ^b^Corresponding expected number of 96 independent populations founded by specified number of cells to 1 decimal place (giving a total of 53.9 wells), and ^d^rounded to the nearest number of wells (54 wells).

The starting inoculum contained roughly a 0.69 proportion of Cap^+^ cells (345/500). Thus, the probability that all 8 cells that were lone founders were capsulated (Pcap) is 0.051, while the probability that all 1 cell populations were founded by non-capsulated cells (Pnoncap) is 8.5x10^-5^. Then, the probability that each cell type founded at least one population (Pboth) is 0.9489. These results are consistent with JG59 cells being capable of switching in both directions.

$Pcap=\left( {0.69}^{8} \right)=0.051$

$$Pnoncap=\left( {0.31}^{8} \right)=0.000085$$

$P\left( both \right)= 1-\left( Pcap+ Pnoncap \right)=0.9489$

*96 well plate for JG71 (Re9)*

The 96 well plate for JG71 showed 25 wells with growth (shaded grey below), and 71 wells with no growth (unshaded). India ink staining followed by bright field microscopy showed a good mix of Cap^+^ and Cap^-^ cells in each well that grew:

|  | 1 | 2 | 3 | 4 | 5 | 6 | 7 | 8 | 9 | 10 | 11 | 12 |
| --- | --- | --- | --- | --- | --- | --- | --- | --- | --- | --- | --- | --- |
| A |  |  |  |  |  |  |  |  |  |  |  |  |
| B |  |  |  |  |  |  |  |  |  |  |  |  |
| C |  |  |  |  |  |  |  |  |  |  |  |  |
| D |  |  |  |  |  |  |  |  |  |  |  |  |
| E |  |  |  |  |  |  |  |  |  |  |  |  |
| F |  |  |  |  |  |  |  |  |  |  |  |  |
| G |  |  |  |  |  |  |  |  |  |  |  |  |
| H |  |  |  |  |  |  |  |  |  |  |  |  |

It is known that there are 71 wells that were “inoculated” with 0 cells (no growth). Thus, we can use the Poisson distribution to calculate *λ,* the expected number of cells per well (*P*=probability of *y* cells founding any given well, *y*=number of cells of interest, *λ*=the expected number of cells *per* well, *e*=base of the natural logarithm):

$\frac{71}{96}=e^{-\lambda}$

$\lambda= -(\ln\left( \frac{71}{96} \right))$ = 0.3016683144265208622

This value of lambda was used to work out the theoretical occurrence of each number of cells inoculating a well (**Table 1.6**):

| **Number of cells** | **Actual or theoretical occurrence probability^a^** | **Number of wells^b^** | |
| --- | --- | --- | --- |
|  |  | **1 d.p.^c^** | **Rounded^d^** |
| 0 | 71/96=0.5520833333 | (53) | 53 |
| 1 | 0.223 | 21.4 | 21 |
| 2 | 0.0337 | 3.2 | 3 |
| 3 | 0.00338 | 0.3 | 0 |
| 4+ | 0.000337 | 0 | 0 |
| Totals | 1.00 | 95.9 | 95 |

**Table 1.6: Poisson probabilities of population foundation by increasing numbers of JG71 (Re9) cells, and corresponding expected numbers of each in 96 independent populations. ^a^**Probability of the specified number of cells founding any given population. ^b^Corresponding expected number of 96 independent populations founded by specified number of cells to 1 decimal place (giving a total of 95.9 wells), and ^d^rounded to the nearest number of wells (95 wells).

The starting inoculum contained roughly a 0.1632 proportion of Cap+ cells. Thus, the probability that all 32 cells that were lone founders were capsulated (Pcap) is 2.93x10^-17^, while the probability that all 1 cell populations were founded by non-capsulated cells (Pnoncap) is 0.0237. Then, the probability that each cell type founded at least one population (Pboth) is 0.9763. These results are consistent with JG71 cells being capable of switching in both directions.

$Pcap=\left( {0.1632}^{21} \right)=2.93x{10}^{-17}$

$$Pnoncap=\left( {0.8368}^{21} \right)=0.02371674597$$

$P\left( both \right)= 1-\left( Pcap+ Pnoncap \right)=0.97628325402$

**Conclusion**

Of the 15 potential switcher genotypes, nine have been recorded as bidirectional Cap^+^/Cap^-^ switchers in the manuscript – on the basis of colony, cell and population growth assays. Each of the nine switcher genotypes contains one of three different point mutations in *rpoD*; the six discarded genotypes contain mutations in *pflu4939* or *wspR*.

**Supplementary Text S2**

**Extended experimental procedures**

**2.1 Bacterial strains used**

| **Name** | **Description** | **Reference** |
| --- | --- | --- |
| SBW25 | Original, wild type strain; starting strain for the Line 6 and Line 1 evolutionary series. | (Rainey and Bailey 1996) |
| *Strains in the Line 6 evolutionary series* | | |
| 6B^0^ | Contains mutation 1 (*wspF* ∆t475, causing a frame shift and altering amino acids from S159 (41 new residues until stop codon reached)). | This study |
| 6A^1^ | Isogenic to SBW25; contains mutation 1 and 2 (*wspF* 474ins.t, an exact reversal of mutation 1, reverting to the wild type genotype). | This study |
| 6B^1^ | Contains mutation 3 (*awsX* ∆229-261, causing the in-frame deletion of amino acids 77-87). | This study |
| 6A^2^ | Contains mutations 3 and 4 (*awsR* c691t, converting amino acid Q231 into a STOP). | This study |
| 6B^2^ | Contains mutations 3, 4 and 5 (*wspF* ∆151-165, causing an in-frame deletion of amino acids 51-55 (∆LMDLI)). | This study |
| 6A^3^ | Contains mutations 3-5 and 6 (*wssB* ∆1720-1725, causing an in-frame deletion of amino acids 574-575 (∆VA)) | This study |
| 6B^3^ | Contains mutations 3-6 and 7 (*nlpD* c565t, converting amino acid Q189 into a STOP). | This study |
| 6A^4^ | Immediate ancestor of the switcher genotype; contains mutations 3-7 and 8 (*nlpD* a566g, converting the STOP codon from 6B^3^, STOP189, to a W). | This study |
| 6B^4^ | Switcher genotype; contains mutations 3-8 and 9 (*rpoD* t1682c, causing amino acid change T1682C). | This study |
| *Strains in the Line 1 evolutionary series* | | |
| 1B^0^ | Contains mutation 1 (*mwsR* g2778a, causing amino acid change M926I). | (Beaumont et al. 2009; Gallie et al. 2015) |
| 1A^1^ | Contains mutation 1 and 2 (*mwsR* g2383a, causing amino acid change E795K). | (Beaumont et al. 2009; Gallie et al. 2015) |
| 1B^1^ | Contains mutation 1, 2 and 3 (*awsX* ∆229-261, causing the in-frame deletion of amino acids 77-87). | (Beaumont et al. 2009; Gallie et al. 2015) |
| 1A^2^ | Contains mutation 1-3 and 4 (*awsR* a1141c, causing amino acid change T381S). | (Beaumont et al. 2009; Gallie et al. 2015) |
| 1B^2^ | Contains mutation 1-4 and 5 (*wspF* 157insG, causing a frame shift and altering amino acid sequence from M52 (236 new residues until stop codon reached). | (Beaumont et al. 2009; Gallie et al. 2015) |
| 1A^3^ | Contains mutation 1-5 and 6 (*wssA* 164insA, causing a frame shift and altering amino acid sequence from S54 (28 new residues until stop codon reached). | (Beaumont et al. 2009; Gallie et al. 2015) |
| 1B^3^ | Contains mutation 1-6 and 7 (*mwsR* c3094g, causing amino acid change R1032G). | (Beaumont et al. 2009; Gallie et al. 2015) |
| 1A^4^ | Immediate ancestor of the switcher genotype; contains mutation 1-7 and 8 (*mwsR* ∆c2553, causing a frame shift and altering amino acid sequence from D851 (2 new residues until stop codon reached). | (Beaumont et al. 2009; Gallie et al. 2015) |
| 1B^4^ | Switcher genotype; contains mutation 1-8 and 9 (*carB* c2020t, causing amino acid change R674C). | (Beaumont et al. 2009; Gallie et al. 2015) |
| *Switcher strains independently isolated from 6A^4^ (all containing mutations 1-8 and 1 novel mutation)* | | |
| Re1 | Switch-causing mutation is *rpoD* a1723c causing amino acid change T575P. | This study |
| Re2 | Switch-causing mutation is *rpoD* a1723c causing amino acid change T575P. | This study |
| Re3 | Switch-causing mutation is *rpoD* a1723c causing amino acid change T575P. | This study |
| Re4 | Switch-causing mutation is *rpoD* a1723c causing amino acid change T575P. | This study |
| Re5 | Switch-causing mutation is *rpoD* a1723c causing amino acid change T575P. | This study |
| Re6 | Switch-causing mutation is *rpoD* a1723c causing amino acid change T575P. | This study |
| Re7 | Switch-causing mutation is *rpoD* a1723c causing amino acid change T575P. | This study |
| Re8 | Switch-causing mutation is *rpoD* a1723c causing amino acid change T575P. | This study |
| Re9 | Switch-causing mutation is *rpoD* a1745c causing amino acid change Q582P. | This study |
| *Reconstructions, deletions and fusions* | | |
| 6A^4^-*rpoD** | 6A^4^ containing a scar-free reconstruction of the *rpoD* t1682c switch-causing mutation (*i.e.*, reconstructed 6B^4^). | This study |
| SBW25-*rpoD** | SBW25 containing a scar-free reconstruction of the *rpoD* t1682c switch-causing mutation. | This study |
| 1A^4^-*rpoD** | 1A^4^ (the immediate ancestor of the Line 1 switcher) containing a scar-free reconstruction of the *rpoD* t1682c switch-causing mutation. | This study |
| 6A^4^-*carB** | 6A^4^ (the immediate ancestor of the Line 6 switcher) containing a scar-free reconstruction of the *carB* c2020t switch-causing mutation. | This study |
| 6A^4^-∆CA | 6A^4^ with a complete, scar-free deletion of the CAP biosynthetic locus (*wcaJ-wzb*). | This study |
| 6B^4^-∆CA | 6B^4^ with a complete, scar-free deletion of the CAP biosynthetic locus (*wcaJ-wzb*). | This study |
| 1A^4^-∆CA | 1A^4^ with a complete, scar-free deletion of the CAP biosynthetic locus (*wcaJ-wzb*). | This study |
| 1B^4^-∆CA | 1B^4^ with a complete, scar-free deletion of the CAP biosynthetic locus (*wcaJ-wzb*). | (Gallie et al. 2015) |
| 6B^4^-∆*rsmA1* | 6B^4^ with a scar-free deletion of *rsmA1* (*pflu4746*). | This study |
| 6B^4^-∆*rsmE* | 6B^4^ with a scar-free deletion of *rsmE* (*pflu4165*). | This study |
| 6B^4^-∆*mvaT* | 6B^4^ with a scar-free deletion of *mvaT* (*pflu4939*). | This study |
| 1B^4^-∆*mvaT* | 1B^4^ with a scar-free deletion of *mvaT* (*pflu4939*). | This study |
|  |  |  |
| 6B^4^-TnCre-*pflu3656* | Cre-deletion of transposon insertion strain 6B4-Tn-5.42 (Table_S1). Contains 189 bp insertion in *pflu3656.* | This study |
| 6B^4^-TnCre-*pflu3657* | Cre-deletion of transposon insertion strain 6B4-Tn-8.10 (Table_S1). Contains 189 bp insertion in *pflu3657.* | This study |
| 6B^4^-TnCre-*gacA* | Cre-deletion of transposon insertion strain 6B4-Tn-5.27 (Table_S1). Contains 189 bp insertion in *gacA (pflu2189).* | This study |
| 6B^4^-TnCre-*gacS* | Cre-deletion of transposon insertion strain 6B4-Tn-6.22 (Table_S1). Contains 189 bp insertion in *gacS (pflu3777).* | This study |
| 6B^4^-TnCre-*truA* | Cre-deletion of transposon insertion strain 6B4-Tn-2.17 (Table_S1). Contains 189 bp insertion in *truA (pflu4189).* | This study |
| 6B^4^-TnCre-*gidA* | Cre-deletion of transposon insertion strain 6B4-Tn-6.13 (Table_S1). Contains 189 bp insertion in *gidA (pflu6129).* | This study |
| 6B^4^-TnCre-*thiI* | Cre-deletion of transposon insertion strain 6B4-Tn-4.24 (Table_S1). Contains 189 bp insertion in *thiI (pflu0349).* | This study |
| *Escherichia coli strains used during molecular genetics* | | |
| *E. coli* DH5α-λ*pir* | supE44, ∆lacU169, *hsdR17*, *recA1*, *endA1*, *gyrA96*, thi- 1, *relA1*,λ*pir* | Invitrogen |
| *E. coli* TOP10 | F', *mcrA*, Δ(mrr-hsdRMS-mcrBC), Φ80lacZΔM15, ΔlacX74, deoR, *recA1*, *araD139*, Δ(ara-leu)7697, *galU*, *galK,* *rpsL*, Str^R^, *endA1*, *nupG* | Invitrogen |

**Table 2.1: Designations and characteristics of bacterial strains used in this study.**

**2.2 Plasmids and transposons used**

| **Name** | **Description** | **Reference** |
| --- | --- | --- |
| *Plasmids* | | |
| pCR8/GW/TOPO | Spe^R^, Puc *ori*; 2.8 kb sequencing plasmid | Invitrogen |
| pUIC3 | Tc^R^, *mob*, *oriR6K*, *bla*, Δpromoter-*lacZY* | (Rainey 1999) |
| pRK2013 | Km^R^, IncP4, *tra*, *mob*; mobilization plasmid used as a helper for triparental mating | (Figurski and Helinski 1979) |
| pCre | A derivative of pUT, carrying the *cre* gene from pRH133, Cm^R^, used to excise IS-Ω-Km/hah, leaving a 189 bp segment with STOP codons in all three reading frames. | (Manoil, 2000; Giddens *et al*., 2007) |
| *Transposons* | | |
| IS-Ω-Km/hah | Km^R^, ColE1 *ori*, *npt* promoter, *loxP* | (Giddens et al. 2007) |

**Table 2.2: Designations and characteristics of plasmids and transposons used in this study.**

**2.3 Genotype constructions**

*2.3.1 Construction of* rpoD *mutations*

The t1682c *rpoD* mutation was reconstructed in three backgrounds: 6A^4^, 1A^4^ and SBW25. Primers used are in the Table below. In each case, the mutation was constructed using a scar-free, pUIC3-mediated two-step allelic exchange protocol (Zhang and Rainey 2007). The resulting genotypes, 6A^4^-*rpoD**, 1A^4^-*rpoD** and SBW25-*rpoD** were confirmed as containing the *rpoD* mutation by PCR amplification and Sanger sequencing of the relevant genomic region.

| **Primer Name** | **Sequence (5**'**🡪3**'**)^a^** | **Use** |
| --- | --- | --- |
| rpoD_t1682c_f | gaagatctGCAGATGTTGCAGGAAATGGGTC | Construction of t1682c |
| rpoD_t1682c_r | gaagatctCCGTTGCGGTCCAGGTC | Construction of t1682c |
| rpoD_final_f | CTGGTGGATTCGTCAGGCGATC | Final PCR & sequencing |
| rpoD_final_r | GCCGTCTTCGTCACGGTATTC | Final PCR & sequencing |

^a^underline denotes *Bgl*II restriction site. Capital letters denote homology to the SBW25 genome sequence, and small letters denote primer sequence that does not match SBW25 (and are not present in the final construct).

*2.3.2 Construction of* carB *mutation*

The c2020t *carB* mutation was reconstructed in 6A^4^. Primers used are in the Table below. The mutation was constructed using a scar-free, pUIC3-mediated two-step allelic exchange protocol (Zhang and Rainey 2007). The resulting genotype, 6A^4^-*carB** was confirmed as containing the c2020t *carB* mutation by PCR amplification and Sanger sequencing of the relevant genomic region.

| **Primer Name** | **Sequence (5**'**🡪3**'**)^a^** | **Use** |
| --- | --- | --- |
| carB_3f | gaagatctTGGGTGTGACCGAGAAGAACC | Construction of c2020t |
| carB_4r | gaagatctCGGGTTGACTTCGATGACGTAG | Construction of c2020t |
| carB_final_f | GTTCCTGGTGCAGATCGAGAGTC | Final PCR & sequencing |
| carB_final_r | CACGCTGTAGAAGTTCGGAATG | Final PCR & sequencing |
| carB_seq_f | CTTCCGACCGTCTGTACTTTGAG | Sequencing |
| carB_seq_f2 | CTGGACCACTTCCTCAACTGC | Sequencing |

^a^underline denotes *Bgl*II restriction site. Capital letters denote homology to the SBW25 genome sequence, and small letters denote primer sequence that does not match SBW25 (and are not present in the final construct).

*2.3.3 Deletion of the colanic acid operon*

The CAP structural gene locus, *wcaJ-wzb* (which covers bases 4050846-4074200), was deleted from 6B^4^ (giving 6B^4^∆CA), using the same set of primers and process as previously published for the 1B^4^ genetic background (Gallie et al. 2015). In short, bases 4050675-4074194 inclusive were replaced with a 23 bp sequence. Sequences of the primers used are provided in the table below.

| **Primer Name** | **Sequence (5**'**🡪3**'**)^a^** | **Use** |
| --- | --- | --- |
| wcaJKO-1 | gaagatctcgcagacgctgaacacctg | Deletion of *wcaJ-wzb* |
| wcaJKO-2 | cagcatgcggatccgttgacggacgtgtgttgataggcagggtg | Deletion of *wcaJ-wzb* |
| wcaJKO-5 | tccgtcaacggatccgcatgctgGAACAAGGCAACCTCCCACG | Deletion of *wcaJ-wzb* |
| wcaJKO-6 | gaagatctCTTTCTCAGCGCGTAACTGCG | Deletion of *wcaJ-wzb* |

^a^underline denotes *Bgl*II restriction site. Capital letters denote homology to the SBW25 genome sequence, and small letters denote primer sequence that does not match the SBW25 genome sequence.

*2.3.4 Deletion of* rsmA1 *and* rsmE *from 6B^4^*

The *rsmA1* (*pflu4746*) and *rsmE* (*pflu4165*) genes were individually deleted from 6B^4^ using the scar free two step allelic exchange method. Despite repeated attempts, we were not able to simultaneously delete *rsmA1* and *rsmE* in the 6B^4^ background. The primers used to construct the deletion fragments have been previously published for deletion of these genes in the 1B^4^ background (Remigi *et al*., 2019).

*2.3.5 Deletion of* mvaT *from 6B^4^ and 1B^4^*

The *mvaT* (*pflu4939*) gene was deleted from 6B^4^ and 1B^4^ using the scar free two step allelic exchange method. The primers used to construct the deletion fragments are provided below:

| **Primer Name** | **Sequence (5**'**🡪3**'**)^a^** | **Use** |
| --- | --- | --- |
| oPR80 | tcagactagtGGTGCAGCAAGTCAGCCAG | Deletion of *mvaT* |
| oPR81 | GCAGGGTAGCCCAACCGATCAGAGACATGTGGACTACCT | Deletion of *mvaT* |
| oPR82 | CCACATGTCTCTGATCGGTTGGGCTACCCTGCTG | Deletion of *mvaT* |
| oPR83 | tcagactagtACCGCTCAAACTGGTGCAT | Deletion of *mvaT* |

^a^underline denotes *Spe*I restriction site. Capital letters denote homology to the SBW25 genome sequence, and small letters denote primer sequence that does not match the SBW25 genome sequence. Red=complimentary regions.

*2.3.6 Transcriptional* lacZ *fusion*

To investigate whether CAP expression is controlled at the transcriptional level, a 6B^4^ chromosomal transcriptional fusion was constructed between *lacZ* and the CAP structural gene *wcaJ*. This fusion has previously been constructed in the 1B^4^ genomic background, and shown to produce blue and white sectored colonies (Gallie et al. 2015).

The transcriptional *lacZ* fusion was made by amplifying *wcaJ* (covers bases 4050846-4052261; the fusion fragment consisted of bases 4050809-4052265; see Table below for primers) and inserted into the *Pseudomonas* suicide vector pUIC3 directly upstream of promoterless *lacZY*. The constructed vector was inserted into chemically competent *E. coli* DH5α-λ*pir* cells and transferred to 6A^4^, 6B^4^ and 1A^4^ *via* tri-parental conjugation (the helper strain was *E. coli* carrying pRK2013, which contains *tra* and *mob* genes for conjugation). Transconjugants were screened for types that had incorporated the vector into the chromosome (at *wcaJ* *via* homologous recombination) by plating on LB+Tc(25 µg mL^-1^)+NF. NF inhibits *E. coli* growth and selects against the donor/helper strains, while tetracycline inhibits growth of the recipient. Successful transconjugants (NF^R^, Tc^R^) were purified. A complete, functional copy of *wcaJ* remained in the final genome (a requirement for realization of the Cap^+^ phenotype, see **supplementary Table S1** and (Gallie et al. 2015).

| **Primer Name** | **Sequence (5**'**🡪3**'**)^a^** | **Use** |
| --- | --- | --- |
| WcaJ-lacZf | gaagatctGTATTGCGCCGCGTGATC | *CAP*-*lacZ* transcriptional fusions |
| WcaJ-lacZr | gaagatctGCGCTCAGTAGATATCCTTGG | *CAP*-*lacZ* transcriptional fusions |

^a^underline denotes *Bgl*II restriction site. Capital letters denote homology to the SBW25 genome sequence, and small letters denote primer sequence that does not match the SBW25 genome sequence.

**Supplementary Text S3**

**Statistical test for differential expression of ribosomal protein genes in 6B^4^**

There are 53 genes identified as encoding ribosomal structural proteins in the *P. fluorescens* SBW25 genome. 33 are predicted to be associated with the 50S ribosomal subunit, and 20 with the 30S. A full list of these is available in **Supplementary Table S7**. If an increase in the expression of ribosomes is indeed behind switching in 6B^4^, we would expect to see the 53 genes encoding ribosomal structural proteins to be more highly expressed in capsulated strains/morphotypes. While we do see some of these genes with statically significant higher expression in 6B^4^-Cap^+^ (vs 6A^4^), many have not been identified as showing statistically significant changes in expression. However, when looking at the expression levels of the genes that do not show statistically significant differences, we see apparent patterns. Here, we seek to explore whether these patterns are significant. It is possible that the noisy nature of the Line 6 RNA-seq replicates has led to the over-zealous exclusion of genes.

**3.1 Premise**

Imagine flipping a coin 53 times, and recording “heads” or “tails” for every flip. If the coin is fair, the probability of “heads” at each flip is 0.5. Using this information, we can calculate the probability of receiving a particular number of heads for one run of the 53-trial experiment. This would be done using the binomial distribution, which assigns “success” (“heads”) or “failure” (“tails”) to each run of the experiment. The parameters are *N* (number of trials) and *p* (probability of success at each trial). Here, *B(N*=53, *p*=0.5).

The probability of (*e.g.*) 40 heads on 53 trials of a fair coin, using either a two or one-tailed test in *R*:

> binom.test(40,53,(1/2),alternative="two.sided")

Exact binomial test

data: 40 and 53

number of successes = 40, number of trials = 53, p-value =

0.0002685

alternative hypothesis: true probability of success is not equal to 0.5

95 percent confidence interval:

0.6171753 0.8624488

sample estimates:

probability of success

0.754717

> binom.test(40,53,(1/2),alternative="greater")

Exact binomial test

data: 40 and 53

number of successes = 40, number of trials = 53, p-value =

0.0001343

alternative hypothesis: true probability of success is greater than 0.5

95 percent confidence interval:

0.638574 1.000000

sample estimates:

probability of success

0.754717

> binom.test(40,53,(1/2),alternative="less")

Exact binomial test

data: 40 and 53

number of successes = 40, number of trials = 53, p-value = 1

alternative hypothesis: true probability of success is less than 0.5

95 percent confidence interval:

0.0000000 0.8483866

sample estimates:

probability of success

0.754717

**3.2 The ribosomal structural genes from the RNA-seq data**

By extrapolation, we can use the above to test the probability of obtaining the observed numbers of higher/lower RNA-seq BaseMeans for the 53 ribosomal structural genes in each morphotype comparison. We will label “increased expression in morphotype 1” as “success” and “increased expression in morphotype 2” as “failure”.

Note that, unlike the coin toss example above, *p* is not necessarily 0.5 in our data. That is, the probability of success/increased expression in morphotype 1 vs morphotype 2 is not necessarily 0.5. Instead, we have looked at the RNA-seq output, and for each comparison determined the number of genes that are increased in expression in morphotype 1, and the number in morphotype 2. These values have then been used to calculate *p* in each case (**Table 3.2.1**). *N*, however, remains 53 (due to there being 53 genes encoding ribosomal structural proteins).

| **Comparison** | **Morphotype 1** | **Morphotype 2** | **# Gene up 1** | **# Genes up 2** | ***p* (4 s.f.)** |
| --- | --- | --- | --- | --- | --- |
| A | 6A^4^ | 6B^4^-Cap^-^ | 3211 | 2799 | 0.5343 |
| B | 6A^4^ | 6B^4^-Cap^+^ | 2919 | 3092 | 0.4856 |
| C | 6B^4^-Cap^-^ | 6B^4^-Cap^+^ | 2559 | 3451 | 0.4258 |

**Table 3.2.1:** The probability of “success” (“increased expression in morphotype 1”) in each comparison.

For example, if we take comparison B (6A^4^ vs 6B^4^-Cap^+^), *H_0_*=no difference in ribosomal structural gene expression between 6A^4^ and 6B^4^-Cap^+^. The question is, given that the chance of any randomly assigned gene showing increased expression in 6A^4^ is 0.4856, what are the odds of 13 (of 53) ribosomal structural genes being increased in 6A^4^ relative to 6B^4^-Cap^+^? A two-tailed binomial test (*N*=53, *p*=2919/6011, success=13) provides strong evidence against *H_0_* (*p*=0.0004791; **Table 3.2.2**).

| **Comparison** | **Morphotype 1** | **Morphotype 2** | **# Success** | **# Failure** | ***p*-value** |
| --- | --- | --- | --- | --- | --- |
| A | 6A^4^ | 6B^4^-Cap^-^ | 29 | 24 | 0.8912 |
| B | 6A^4^ | 6B^4^-Cap^+^ | 13 | 40 | 0.0004791*** |
| C | 6B^4^-Cap^-^ | 6B^4^-Cap^+^ | 10 | 43 | 0.0004082*** |

**Table 3.2.2:** Summary of “successes” for the 53 ribosomal structural genes in SBW25. A significant *p*-value provides evidence against *H_0_* (no difference in expression of ribosomal structural genes in morphotype 1 and 2).

The one tailed test with *H_1_* of *p*<0.4856 has a significant *p*-value (*p=*0.0002929; **Table 3.2.3**), providing strong evidence that *p* is less than 0.4856 for the ribosomal structural genes. The estimate of the true *p* for ribosomal structural genes in this comparison is 0.2453, with 95% CI of 0-0.3614. In other words, the chance of “success” or “higher expression in 6A^4^ than 6B^4^-Cap^+^” is 0.2453.

| **Comp** | **Morph 1** | **Morph 2** | **One tailed test *H_1_*** | ***p*-value** | **Higher expression morphotype** | **Estimate true *p* (4 s.f.)** | **95% CI for *p* (4 s.f.)** |
| --- | --- | --- | --- | --- | --- | --- | --- |
| A | 6A^4^ | 6B^4^-Cap^-^ | n/a | n/a | n/a | n/a | n/a |
| B | 6A^4^ | 6B^4^-Cap^+^ | *p*<0.4856 | 0.0002929 | 6B^4^-Cap^+^ | 0.2453 | 0-0.3614 |
| C | 6B^4^-Cap^-^ | 6B^4^-Cap^+^ | *p*<0.4258 | 0.0002356 | 6B^4^-Cap^+^ | 0.1887 | 0-0.2990 |

**Table 3.2.3:** Summary of one-tailed tests to determine direction of preferred over-expression.

**3.3 R (version 3.3.3) output**

*Comparison A: 6A^4^ vs 6B^4^-Cap^-^*

*H_0_*=no difference in expression of 53 ribosomal structural genes between 6A^4^ and 6B^4^-Cap^-^. If *H_0_* holds, what are the odds of getting 29 “success” (“up in morphotype 1”), when *N=*53 and *p*=3211/6010?

> binom.test(29,53,(3211/6010),alternative="two.sided")

Exact binomial test

data: 29 and 53

number of successes = 29, number of trials = 53, p-value = 0.8912

alternative hypothesis: true probability of success is not equal to 0.5342762

95 percent confidence interval:

0.4044983 0.6843847

sample estimates:

probability of success

0.5471698

> binom.test(29,53,(3211/6010),alternative="less")

Exact binomial test

data: 29 and 53

number of successes = 29, number of trials = 53, p-value = 0.6265

alternative hypothesis: true probability of success is less than 0.5342762

95 percent confidence interval:

0.0000000 0.6647034

sample estimates:

probability of success

0.5471698

> binom.test(29,53,(3211/6010),alternative="greater")

Exact binomial test

data: 29 and 53

number of successes = 29, number of trials = 53, p-value = 0.4812

alternative hypothesis: true probability of success is greater than 0.5342762

95 percent confidence interval:

0.4255312 1.0000000

sample estimates:

probability of success

0.5471698

Conclusion: no evidence against equal expression of ribosomal structural protein encoding genes in 6A^4^ and 6B^4^-Cap^-^ (*p*>0.5).

*Comparison B: 6A^4^ vs 6B^4^-Cap^+^*

*H_0_*=no difference in expression of 53 ribosomal structural genes between 6A^4^ and 6B^4^-Cap^+^. If *H_0_* holds, what are the odds of getting 13 “success” (“up in morphotype 1”), when *N=*53 and *p*=2919/6011?

> binom.test(13,53,(2919/6011),alternative="two.sided")

Exact binomial test

data: 13 and 53

number of successes = 13, number of trials = 53, p-value =

0.0004791 ***

alternative hypothesis: true probability of success is not equal to 0.4856097

95 percent confidence interval:

0.1375512 0.3828247

sample estimates:

probability of success

0.245283

> binom.test(13,53,(2919/6011),alternative="less")

Exact binomial test

data: 13 and 53

number of successes = 13, number of trials = 53, p-value =

0.0002929 ***

alternative hypothesis: true probability of success is less than 0.4856097

95 percent confidence interval:

0.000000 0.361426

sample estimates:

probability of success

0.245283

> binom.test(13,53,(2919/6011),alternative="greater")

Exact binomial test

data: 13 and 53

number of successes = 13, number of trials = 53, p-value = 0.9999

alternative hypothesis: true probability of success is greater than 0.4856097

95 percent confidence interval:

0.1516134 1.0000000

sample estimates:

probability of success

0.245283

Conclusion: strong evidence against equal expression of ribosomal structural genes in 6A^4^ and 6B^4^-Cap^+^ (*p*<0.001). The one tailed test with *H_1_* of *p*<0.4856 has a significant *p*-value (*p=*0.0002929), providing strong evidence that *p* is less than 0.4856 for the ribosomal structural genes. The estimate of the true *p* for ribosomal structural genes in this comparison is 0.2453, with 95% CI of 0-0.3614. In other words, the chance of “success” or “higher expression in 6A^4^ than 6B^4^-Cap^+^” for any given trial is 0.2453.

Note also that we would expect the biggest difference in expression between morphotypes 6A^4^ vs 6B^4^-Cap^+^. In this comparison, 3 of the 40 ribosomal structural protein genes that are more highly expressed in 6B^4^-Cap^+^ show a statistically significant difference (**Table 3.3**):

| **#** | **Gene** | **Pflu** | **Product** | **Fold change** | **Adj. *p*-value** |
| --- | --- | --- | --- | --- | --- |
| 1 | *rpmB* | *pflu5980* | 50S ribosomal protein L28 | 7.738548414 | 0.00020082 |
| 2 | *rpmG* | *pflu5979* | 50S ribosomal protein L33 | 8.319071726 | 0.002195541 |
| 3 | *rpsT* | *pflu0765* | 30S ribosomal protein S20 | 4.143637957 | 0.037381413 |

**Table 3.3: Genes encoding ribosomal structural proteins that show statistically significantly higher expression in 6B^4^-Cap^+^ than 6A^4^ (Supplementary table S4).**

*Comparison C: 6B^4^-Cap^-^ vs 6B^4^-Cap^+^*

*H_0_*=no difference in expression of 53 ribosomal structural genes between 6B^4^-Cap^-^ and 6B^4^-Cap^+^. If *H_0_* holds, what are the odds of getting 10 “success” (“up in morphotype 1”), when *N=*53 and *p*=2559/6010?

> binom.test(10,53,(2559/6010),alternative="two.sided")

Exact binomial test

data: 10 and 53

number of successes = 10, number of trials = 53, p-value =

0.0004082***

alternative hypothesis: true probability of success is not equal to 0.4257903

95 percent confidence interval:

0.09436978 0.31972435

sample estimates:

probability of success

0.1886792

> binom.test(10,53,(2559/6010),alternative="less")

Exact binomial test

data: 10 and 53

number of successes = 10, number of trials = 53, p-value =

0.0002356***

alternative hypothesis: true probability of success is less than 0.4257903

95 percent confidence interval:

0.0000000 0.2989995

sample estimates:

probability of success

0.1886792

> binom.test(10,53,(2559/6010),alternative="greater")

Exact binomial test

data: 10 and 53

number of successes = 10, number of trials = 53, p-value = 0.9999

alternative hypothesis: true probability of success is greater than 0.4257903

95 percent confidence interval:

0.1060974 1.0000000

sample estimates:

probability of success

0.1886792

Conclusion: strong evidence against equal expression of ribosomal structural genes in 6B^4^-Cap^-^ and 6B^4^-Cap^+^ (*p*<0.001). The one tailed test with *H_1_* of *p*<0.4258 has a significant *p*-value (*p=*0.0002356), providing strong evidence that *p* is less than 0.4258 for the ribosomal structural genes. The estimate of the true *p* for ribosomal structural genes in this comparison is 0.1887, with 95% CI of 0-0.2990. In other words, the chance of “success” or “higher expression in 6B^4^-Cap^-^ than 6B^4^-Cap^+^” on any given trial is 0.1887.

**Supplementary Figure S1**

**
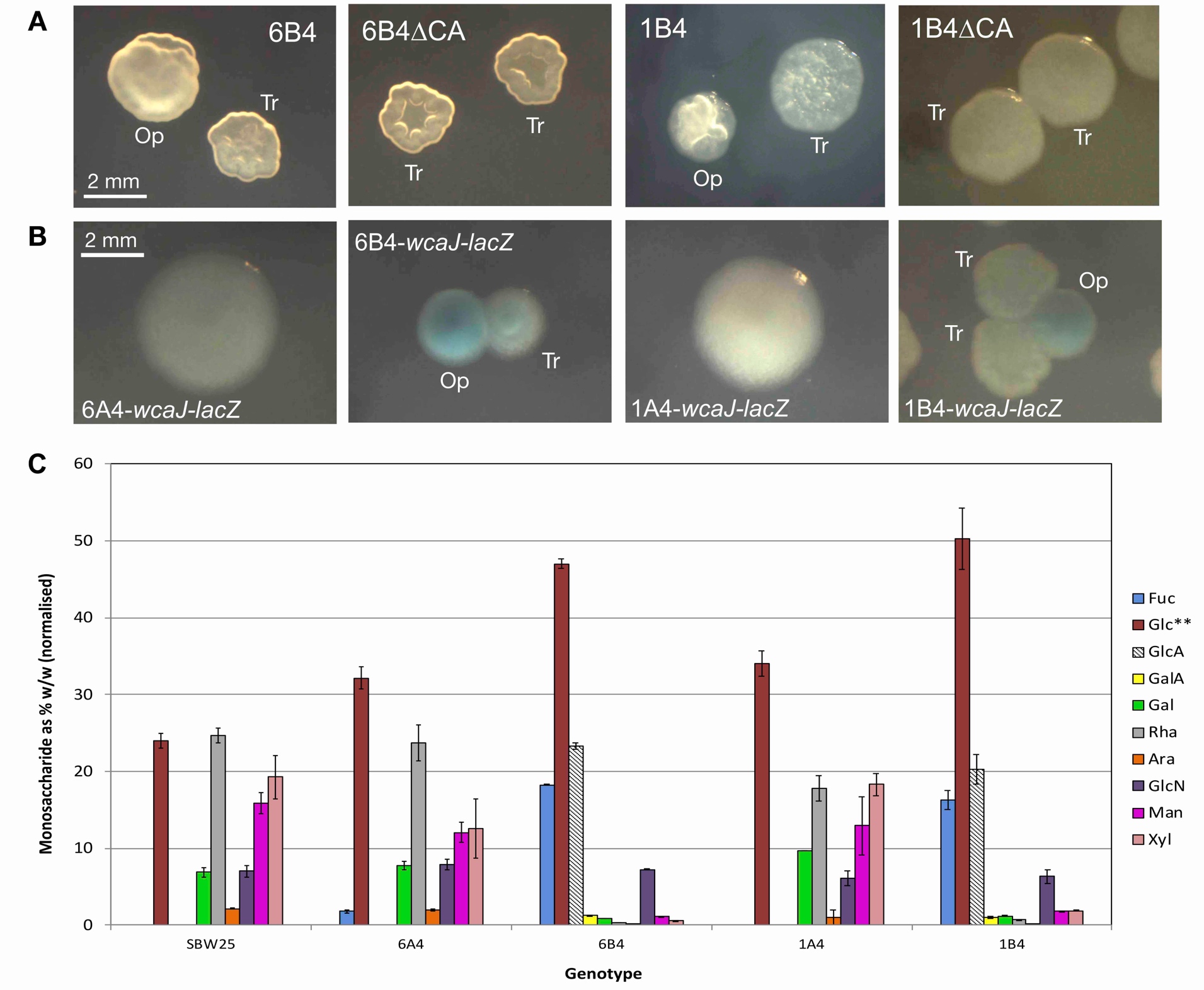
**

**Supplementary figure S1. The structural basis of the capsule is a colanic acid-like polymer (CAP).** (**A**) Deletion of *wcaJ-wzb* (the ~23.3 kb CAP-biosynthetic locus) from 6B^4^ and 1B^4^ results in loss of colony bistability (no opaque colonies). (**B**) Colonies in which a transcriptional fusion of *lacZ* has been made to CAP biosynthetic gene *wcaJ* in the 6A^4^, 6B^4^, 1A^4^ and 1B^4^ backgrounds (on LB+Tc+X-gal plates, 42 hours). 6B^4^-*wcaJ-lacZ* and 1B^4^*-wcaJ-lacZ* immediately develop a mixture of blue (Op) and white (Tr) colonies, showing a degree of transcriptional control of *wcaJ*. Contrastingly, 6A^4^-*wcaJ-lacZ* and 1A^4^-*wcaJ-lacZ* initially form uniform, white colonies. Many of these go on to develop a blue tinge, indicating an increase in *wcaJ* transcription at higher population densities and/or later growth phases. This is consistent with the observation that all ancestral genotypes tested carry a low level of capsulated cells, despite producing uniform colonies (Gallie *et al* ., 2015). (**C**) Analysis of the monosaccharide components of all extracellular polysaccharide produced by 6B^4^, 1B^4^ and ancestors shows the emergent production of a similar polymer in both 6B^4^ and 1B^4^ (data for SBW25, 1A^4^ and 1B^4^ published previously in Gallie *et al*., 2015; presented here for comparison). There are two unidentified sugar components found in 6B^4^ and 1B^4^ (not shown). Bars=mean of 2 replicate measurements, error bars=1 SE (see also **supplementary Table S2**). Contrast and/or exposure of some images altered in Preview.

**Supplementary Tables (legends)**

This section contains the legends for seven supplementary tables available as excel files.

**Supplementary text S1. Characterization of capsulation in Line 6 switcher genotypes.** Measurement of capsule size in 6B^4^ versus 1B^4^; demonstration of bi-directional ON/OFF capsule switching in the three *rpoD* mutants (6B^4^, Re8, Re9);

**Supplementary text S2. Extended experimental procedures.** Lists of bacterial strains, plasmids and primers; details of strain constructions.

**Supplementary text S3. Statistical test for differential expression of ribosomal protein genes in 6B^4^.**

**Supplementary table S1. 6B^4^ transposon mutants with altered switching.** Genotypic and phenotypic details of 55 transposon mutants in which the transposon affects capsule switching. Mutants are sorted according to the cellular function likely to be affected by the capsulation-altering insertion. The precise genomic location of the transposon is indicated as the first base on the genomic forward strand downstream of the 3ʹ transposon terminus. For mutants of particular interest, a Cre-deletion (removing most of the transposon and thus eliminating polar effects; Giddens *et al*. 2007) was obtained and analysed (**fig. 6C**).

**Supplementary table S2. Analysis of the capsule polymer monosaccharide composition.** Analysis of the monosaccharide composition of capsule polymer isolated from SBW25, 6A^4^, 6B^4^, 1A^4^ and 1B^4^ (SBW25, 1A^4^ and 1B^4^ measures presented previously (Gallie *et al*., 2015). The analysis is split into three worksheets. The first presents the dry weight (after freeze-drying) of the polymer sample isolated from each genotype. The second lists the monosaccharide composition of each of two replicates of the polymer samples. Each measurement is presented as % w/w and is expressed as a percentage of the detected sugars (*i.e.*, has been normalised). The third worksheet contains the averages, standard errors, and graphs for the analysis.

**Supplementary table S3. A comparison of gene expression data from 6A^4^ and 6B^4^-Cap^-^ (comparison A).** Comparison showing the effects of mutations 1–8 in the evolutionary series (see **fig. 2A**) on gene expression. Complete dataset available at the National Center for Biotechnology Information (NCBI) Gene Expression Omnibus (GEO) (accession number GSE116490). The table is a list of genes identified by mRNA-seq as having detectably higher mRNA levels in the first genotype (worksheet 1) or the second genotype (worksheet 2). Genes in each worksheet are first sorted into genes showing statistically significant (adjusted *p*<0.05) and not statistically significant (adjusted *p*>0.05) different expression levels (column K), and then by decreasing fold change (column H). Column A is “Locus” (the n^th^ gene in the SBW25 genome; note that while this correlates with pflu number, it is not necessarily the same); column B is “Gene Name” (assigned name or pflu number); column C is “Product” (protein product of the gene); column D is “EC Number” (enzyme catalogue number, where available); column E is “Colour in SBW25 Genome” (colours 1–15 each indicate a distinct functional class of gene product); columns F and G are “Genotype1 BaseMean” and “Genotype2 BaseMean,” respectively (BaseMean is the normalized mean expression level of two replicates of the relevant genotype); column H is “Fold Change” (calculated by dividing the BaseMean of each genotype as indicated); column I is “Log2 Fold Change”; column J is “p-value” (calculated by assuming a binomially distributed read coverage analogous to Fisher’s exact test (Robinson and Smyth, 2008; Anders and Huber, 2010); and column K is “Adjusted p-value” (the p-value adjusted for multiple testing with the Benjamini-Hochberg procedure, which controls for false discovery rate (Anders and Huber, 2010). The third and fourth worksheets provide the raw normalised BaseMeans and a summary of all data in the document, respectively.

**Supplementary table S4. A comparison of gene expression data from 6A^4^ and 6B^4^-Cap^+^ (comparison B).** For full legend see legend of **Supplementary table S3.**

**Supplementary table S5. A comparison of gene expression data from 6B^4^-Cap^-^ and 6B^4^-Cap^+^ (comparison C).** For full legend see legend of **Supplementary table S3.**

**Supplementary table S6. Comparison of Line 6 and Line 1 gene expression data.** mRNA-seq comparisons A (A^4^ versus B^4^-Cap^-^), B (A^4^ versus B^4^-Cap^+^) and C (B^4^-Cap^-^ versus B^4^-Cap^+^) were also performed in Line 1 (GSE48900; Gallie *et al*., 2015). This table compares each of comparisons A, B and C in Line 6 and Line 1. There are six worksheets. Worksheets 1 and 2 concern comparison A, worksheets 3 and 4 comparison B and worksheets 5 and 6 comparison C. The first of each worksheet pair lists all genes with statistically significantly increased expression in the first genotype (those shared by Line 6 and Line 1, followed by those unique to Line 6 and Line 1), and the second of each worksheet pair lists all genes with statistically significantly increased expression in the second genotype (those shared by Line 6 and Line 1, followed by those unique to Line 6 and Line 1). For example, worksheet 1 lists all genes with statistically significantly increased expression in A^4^ versus B^4^-Cap^-^ and worksheet 2 lists all genes with statistically significantly increased expression in B^4^-Cap^-^ versus A^4^.

**Supplementary table S7. Comparison of ribosomal gene expression.** The first worksheet contains a list of all 53 genes in *P. fluorescens* SBW25 that are predicted to encode ribosomal structural proteins. The second worksheet contains a list of all genes encoding ribosomal structural proteins that are differentially expressed in the Line 6 mRNA-seq comparisons A, B and C. Column headings are as described in the legend for **supplementary table S3**. Statistically significant (adjusted *p<*0.05) are highlighted in grey.

**Supplementary References**

Beaumont HJE, Gallie J, Kost C, Ferguson GC, Rainey PB. 2009. Experimental evolution of bet hedging. Nature 462:90–93.

Figurski DH, Helinski DR. 1979. Replication of an origin-containing derivative of plasmid RK2 dependent on a plasmid function provided in trans. Proc. Natl. Acad. Sci. U.S.A. 76:1648–1652.

Gallie J, Libby E, Bertels F, Remigi P, Jendresen CB, Ferguson GC, Desprat N, Buffing MF, Sauer U, Beaumont HJE, et al. 2015. Bistability in a metabolic network underpins the *de novo* evolution of colony switching in *Pseudomonas fluorescens*. PLoS Biol. 13:e1002109.

Giddens SR, Jackson RW, Moon CD, Jacobs MA, Zhang X-X, Gehrig SM, Rainey PB. 2007. Mutational activation of niche-specific genes provides insight into regulatory networks and bacterial function in a complex environment. Proc. Natl. Acad. Sci. U.S.A. 104:18247–18252.

Manoil C, 2000. Tagging exported proteins using *Escherichia coli* alkaline phosphatase gene fusions. Meth. Enzymol. 326:35-47.

Rainey PB, Bailey MJ. 1996. Physical and genetic map of the *Pseudomonas fluorescens* SBW25 chromosome. Mol. Microbiol. 19:521–533.

Rainey PB. 1999. Adaptation of *Pseudomonas fluorescens* to the plant rhizosphere. Environ. Microbiol. 1:243–257.

Remigi P, Ferguson GC, De Monte S, Rainey PB. 2018. Ribosome provisioning activates a bistable switch coupled to fast exit from stationary phase. Available from: https://www.biorxiv.org/content/early/2018/04/24/244129

Zhang X-X, Rainey PB. 2007. Genetic analysis of the histidine utilization (*hut*) genes in *Pseudomonas fluorescens* SBW25. Genetics 176:2165–2176.
